# Elaidic acid suppresses hepatocellular carcinoma growth through modulating the production of intestinal *Ligilactobacillus murinus*-derived spermidine

**DOI:** 10.7150/ijbs.122392

**Published:** 2026-01-14

**Authors:** Yini Li, Tongtong Tian, Qian Yu, Huiqin Jiang, Te Liu, Hao Wang, Ran Huo, Chenzheng Gu, Yu Liu, Ying Zhao, Chunyan Zhang, Yan Zhou, Jiyan Wang, Lin Ding, Chuyu Wang, Xinyi He, Wei Guo, Wenjing Yang, Beili Wang

**Affiliations:** 1 Department of Laboratory Medicine, Zhongshan Hospital, Fudan University, Shanghai, China; 2 Shanghai Geriatric Institute of Chinese Medicine, Shanghai University of Traditional Chinese Medicine, Shanghai, China.; 3 Department of Laboratory Medicine, Wusong Branch, Zhongshan Hospital, Fudan University, Shanghai, China; 4 Department of Laboratory Medicine, Xiamen Branch, Zhongshan Hospital, Fudan University, Xiamen, China; 5 Department of Laboratory Medicine, Shanghai Geriatric Medical Center, Shanghai, China; 6 Cancer Center, Zhongshan Hospital, Fudan University, Shanghai, China

**Keywords:** elaidic acid, hepatocellular carcinoma, *Ligilactobacillus murinus*, spermidine

## Abstract

Dietary intervention provides a novel approach for cancer therapy. Elaidic acid (EA), which accounts for 80-90% of total trans fatty acids in foods, has recently been found to exert anti-tumor effects. However, the biological functions and underlying mechanisms of EA remain elusive in hepatocellular carcinoma (HCC). In this study, targeted fatty acid metabolomics demonstrated that among 44 types of fatty acids, the concentration of EA decreased most significantly when comparing plasma from HCC patients with plasma from healthy people. Through *in vivo* assays using HCC orthotopic and xenograft mouse models, we further revealed that dietary EA attenuates HCC growth. Notably, when gut microbiota was depleted using a cocktail of antibiotics, the anti-tumor effect of EA was diminished, confirming that EA suppresses HCC tumor growth by modulating gut microbiota. Mechanistically, analysis of 16S ribosomal RNA sequencing showed that dietary EA markedly increases the abundance of intestinal *Ligilactobacillus murinus (L. murinus)*. Subsequent untargeted metabolomic sequencing analysis further demonstrated that dietary EA drives the production of *L. murinus*-derived spermidine (SPD), which attenuates HCC growth *in vitro* as well as* in vivo*. The observed impact correlated with the phosphorylation of p38 MAPK and the upregulation of biomarkers pertinent to apoptosis and proliferation, including tumor protein 53, bcl-2-associated X protein, and cysteine-requiring aspartate protease 3. Taken together, our findings highlight the important role of intestinal *L. murinus*-derived SPD in EA-mediated HCC suppression, thereby offering a promising dietary strategy for HCC treatment.

## 1. Introduction

As the predominant primary liver neoplasm, hepatocellular carcinoma (HCC) carries an inferior prognosis 1. The clinical management of HCC presents significant challenges in China, where approximately 70% of individuals receive a diagnosis of moderate or advanced-stage illness at the initial assessment 2. In advanced-stage patients, drug therapy is an indispensable approach. While current therapeutic modalities have improved patient outcomes, their overall efficacy remains suboptimal. Therefore, it is essential to develop innovative approaches for cancer treatment that extend beyond traditional methods.

Dietary therapy is a medical approach that assists in treating tumors by adjusting the dietary structure and components 3. Trans fatty acid (TFA) treatment based on dietary therapy is a promising avenue for suppressing tumorigenesis. TFAs are unsaturated fatty acids characterized by, at a minimum, one unconjugated trans-configured double bond, commonly utilized in the manufacturing of various consumer food products 4. Among all TFAs, elaidic acid (EA, C18:1 trans) constitutes 80-90% of total TFAs in food products 5. EA, as the trans isomer of oleic acid (OA, C18:1 cis), is present in partially hydrogenated vegetable oils and ruminant animal fat. Cooking oil is indispensable in the daily diet, which is consumed by everyone. Therefore, it is of considerable importance to explore the effects of components of vegetable oil on patients with liver cancer. Nevertheless, there has always been controversy regarding the role of EA in tumors. And it has been proved that EA is connected with atherosclerotic cardiovascular disease 6, 7. A previous study demonstrated the significant metastatic capability of colorectal cancer cells treated with EA 8. On the contrary, a recent study revealed that it is possible to impede tumor advancement and improve the efficacy of PD-1 treatment through dietary supplementation of EA 9. However, there is still limited understanding about the effect of EA on liver cancer so far.

Dietary components were associated with tumor progression by impacting the composition and abundance of the intestinal microbes. Metabolites derived from the gut microbiota are important factors influencing tumor growth 10. The gut microbiota modulates essential metabolic, inflammatory, and immune processes, performing critical roles in health and disease. Recent evidence indicates that microbiota significantly influences carcinogenesis and anti-cancer immune responses in both murine models and human patients. 11. The composition of gastrointestinal microbiota and its alteration during disease and treatment affect the outcomes of almost any type of cancer therapy, albeit with different mechanisms 12. In addition, emerging experimental and clinical evidence demonstrated that gut microbiota can be targeted to enhance the effectiveness of cancer treatment. For example, a recent investigation demonstrated that acetate produced by Bifidobacterium pseudolongum inhibits HCC linked with non-alcoholic fatty liver disease 13. However, whether EA modulates the growth of HCC through intestinal microbiota continues to be difficult to define.

This study sought to examine the impact of EA on HCC. Through three kinds of HCC mouse models and an antibiotic-treated mouse model, we demonstrated that the suppression of EA on HCC is related to gut microbiota. Next, 16S rRNA sequencing analysis and untargeted metabolomic sequencing suggested that EA markedly increases the prevalence of *L. murinus* and the concentration of *L. murinus*-derived SPD in the intestinal tract. Surprisingly, our findings revealed the important role of intestinal *L. murinus*-derived SPD in EA-mediated HCC suppression *in vitro* and *in vivo*, which will offer an innovative dietary strategy for HCC treatment.

## 2. Materials and Methods

### 2.1 Animals

All mice were of a Balb/C genetic background. Male mice aged six to eight weeks were acquired from Shanghai GemPharmatech Co., Ltd. and housed under specific pathogen-free (SPF) conditions in Fudan University. The trials were conducted in compliance with the guidelines sanctioned by Fudan University (202312017S), according to the guidelines established by the Institutional Animal Care and Use Committee (IACUC).

### 2.2 Tumor models

For orthotopic tumor models, H22-luc cells (2×10^6^ cells/mL) resuspended in 10 µl phosphate-buffered saline (PBS) (Biosharp, China) were injected into the mice's liver. Tumor burden was quantified by bioluminescence analysis using an *in vivo* imaging system (IVIS).

For subcutaneous xenograft tumor models, H22-luc cells (2×10^7^ cells/mL) resuspended in 100 µl PBS were injected subcutaneously into one flank of the mice. Tumor diameters were assessed with calipers every three days, and the tumor volume was calculated utilizing the formula 14 *0.5 × length × width².* Following a period of 21 days, the mice were anesthetized and sampled. The tumor tissues were measured for weight. Mice were euthanized upon reaching a tumor volume of 2000 mm³ or upon exhibiting humane endpoints, encompassing considerable weight reduction or a hunched back posture.

For tail vein xenograft tumor models, H22-luc cells (1×10^7^ cells/mL) were resuspended in 200 µL PBS and administered by tail vein injection in mice.

For fatty acid supplementation experiments, EA (MCE, #HY-113016) was delivered intragastrically at a dose of 10 mg/kg every day 9. For SPD treatment, mice were administered 3 mM SPD (MCE, HY-B1776) diluted with drinking water.

### 2.3 Antibiotic treatment (Abx)

The gut microbiota in murine intestines were depleted through the administration of an antibiotic cocktail comprising 1 g/L metronidazole (Aladdin, #M109874), 1 g/L ampicillin (Aladdin, #A102048), 1 g/L neomycin (Sangon Biotech, #A610336), and 0.5 g/L vancomycin (Aladdin, #V301569) in water for drinking over a two-week period 15.

### 2.4 Administration of bacteria

*Ligilactobacillus murinus* (SHBCC, D50171) was obtained from the Shanghai Microbiological Culture Collection Center (SHMCC). The bacterial strain was cultured microaerobically in de Man, Rogosa, and Sharpe (MRS) broth in a sealed tube at 37 °C.

Upon reaching an optical density (OD) of 600 at 0.5 for *L. murinus*, the culture medium underwent centrifugation at 12,000 rpm for 20 min, followed by filtering using a 0.22 µm pore-size filter to extract the supernatant of *L. murinus*.

*L. murinus* was subjected to centrifugation and subsequently resuspended in PBS. Mice in the *L. murinus* group received a daily gavage of *L. murinus* at a dosage of 1×10^8^ colony-forming units (CFUs)/mL 16. Mice in the PBS group were administered a gavage of an equivalent volume of sterile PBS every day. Heat-killed *L. murinus* was obtained by heating the bacteria in a metallic bath at 100 °C for 30 minutes 17.

### 2.5 Cell culture

The Liver Cancer Institute at Zhongshan Hospital, Fudan University, provided human HCC cell lines, Huh7 and PLC, cultivated in Dulbecco's Modified Eagle Medium (DMEM, Gibco) with supplementation of 10% fetal bovine serum (FBS) (BIOSUN) and 1% penicillin/streptomycin solution (Gibco). The Chinese Academy of Medical Sciences' Institute of Cell Science provided the mouse HCC cell line H22-luc, which was cultivated in Roswell Park Memorial Institute (RPMI) 1640 medium (Gibco). All cells were cultivated with 5% CO₂ in an incubator at 37 °C (Thermo Fisher, USA).

### 2.6 Cell viability assay

Huh7 and PLC cells (1×10^4^ cells/well) were maintained in 96-well plates. 100 μl of fresh culture medium with EA at doses of 0, 0.25, 0.50, 0.75, 1.00, or 1.25 mM was used to incubate the cells for 24 h.

The Cell Counting Kit-8 (CCK-8) (Beyotime, China) was employed to assess cell viability. After filling each well with 10 μl CCK-8 solution, the plates were incubated at 37°C for 1 h. The absorbance of each sample was quantified at 450 nm with a Sunrise microplate reader (Tecan, Switzerland). As the PLC cell line's half maximum inhibitory concentration (IC_50_) was 221.4 µM and the Huh7 cell line's was 148.6 µM, we used 148.6 µM SPD in the tests that followed.

### 2.7 Colony formation assay

In 6-well plates, cells were inoculated at a density of 1×10^4^ cells per well. One day later, cells were treated with or without 1.25 mM EA, *L. murinus* supernatant, or 148.6 μM SPD for 12 hours. Over two weeks, DMEM medium was replaced every three days. After that, the colonies underwent washing, staining, fixing, and imaging. Software called ImageJ was used to quantify the colonies. In triplicate, the experiments were conducted.

### 2.8 Transwell assay

Cells (3×10^4^) were seeded into the upper chamber (8 μm pore size, Corning, USA) with serum-free DMEM following a 12-hour treatment with 1.25 mM EA, *L.M.* supernatant, or 148.6 μM SPD. Media containing serum was then poured into the lower chamber. Following 48 hours of incubation, the cells were fixed with 4% paraformaldehyde (Biosharp, China) for 15 min, stained for 30 min with crystal violet staining solution (Beyotime, China), rinsed with PBS, and subsequently analyzed. Three duplicates of each experiment were conducted.

### 2.9 Wound-healing assay

PLC and Huh7 cells were wounded using a sterile 200-μl pipette tip after achieving complete confluency in 24-well plates. This was followed by washing with media free of serum. The remaining cells were then subjected to a 12-h co-culture with either 1.25 mM EA, *L.M.* supernatant, or 148.6 μM SPD. The cells were cultured for 48 hours following the medium replacement with DMEM containing 2% FBS. Microscopic images of the wound gap were captured using an Olympus microscope (Japan). ImageJ software was used to quantify the healing areas. The trials were carried out three times.

### 2.10 Cell death detection

In 6-well plates, cells (5×10^5^ per well) were cultivated and treated for 24 h with either 1.25 mM EA, *L.M.* supernatant, or 148.6 μM SPD. Then cells were digested with 0.25% trypsin and collected by centrifugation. The gathered cells were subjected to fixation with 70% ice-cold ethanol for 24 h at 4 °C. Cells were fixed, then washed twice with PBS buffer before being tagged with propidium iodide/Annexin V (Biolegend, USA) and left in the dark for 15 min. FlowJo 10.1 software (USA) was used to examine the results of the assessment of cell apoptosis using a flow cytometer (SONY, Japan). Three duplicates of each experiment were carried out.

### 2.11 Western blot

The enhanced BCA protein assay kit (Beyotime, China) was used to measure the protein content after HCC cells were produced using RIPA buffer. Using 12% sodium dodecyl sulfate-polyacrylamide gel electrophoresis (SDS-PAGE), the total protein was extracted and separated. After being moved onto a nitrocellulose (NC) membrane (Millipore, Germany), the proteins were blocked using Beyotime's QuickBlock buffer. The membranes were washed for 15 min at 20 °C using TBST (Sango Biotech, China). Primary antibodies (1:1000, Affinity, China) were then added to the membranes and incubated for 12 h at 4 °C. The membranes were subsequently treated with HRP-conjugated secondary antibodies (1:1000, Beyotime, China) for 45 min after being cleaned with TBST at 20 °C. They were then washed three times at 20°C and identified utilizing a developer solution (Beyotime, China).

### 2.12 Hematoxylin and eosin (H&E) staining

The tumor tissue underwent fixation, dehydration, embedding, and sectioning. The slices were fixed for 2 min with 95% anhydrous ethanol, then stained with hematoxylin and differentiated for 2 min in a differentiation solution. Following 3 min of immersion in diluted ammonia, the slices were washed with deionized water for 5 min and then stained with eosin for 5 min and washed. For 1 min each, the portions were submerged in ethanol solutions. Following two 1-min washes with anhydrous ethanol, the sections were then given a 1-min xylene wash. The sections were viewed using a light microscope (Olympus, Japan) after being immersed in neutral sesame oil.

### 2.13 Immunohistochemistry

Immunohistochemistry was utilized to evaluate Ki67 expression in hepatic tumor tissue. Following dewaxed paraffin-embedded sections, the slices were incubated in a 3% hydrogen peroxide solution to decrease endogenous peroxidase activity. The slices were incubated with 3% BSA (Servicebio, China) for blocking in order to reduce nonspecific binding. Following that, the slices were treated with primary antibodies Ki67 (1:1000, Abcam). After the main antibodies were incubated, the slices were incubated with a secondary antibody (Servicebio, China) for counterstaining and DAB (DAKO, Denmark) for staining in order to visualize the immunoreactivity.

### 2.14 16S rRNA sequencing

Mouse feces were taken from the EA gavage group (n=5) as well as the DMSO gavage group (n=5). After being extracted from fecal samples, genomic DNA was examined using NanoDrop 2000 and electrophoresis to determine its content and purity. The library construction and sequencing were done by Majorbio Bio-Pharm Technology Co., Ltd. (Shanghai, China).

### 2.15 Metabolomic analysis

Fecal samples are obtained from orthotopic HCC mice that have been treated with DMSO or EA. Fluid samples include *L. murinus* supernatant and MRS medium. To remove contaminants and organisms, the samples of culture supernatant were centrifuged at 14000 g for 10 min. Following hydrochloric acid acidification to pH 2.5, the resultant supernatant was extracted twice using twice as much ethyl acetate. Following air drying, the sample was redissolved in a tenth volume of methanol. Filtration and subsequent testing were performed on the supernatant. The sequencing was done by Majorbio Bio-Pharm Technology Co., Ltd. (Shanghai, China).

## 3. Results

### Result 1: Dietary elaidic acid suppresses HCC *in vivo*

**Figure 1:**
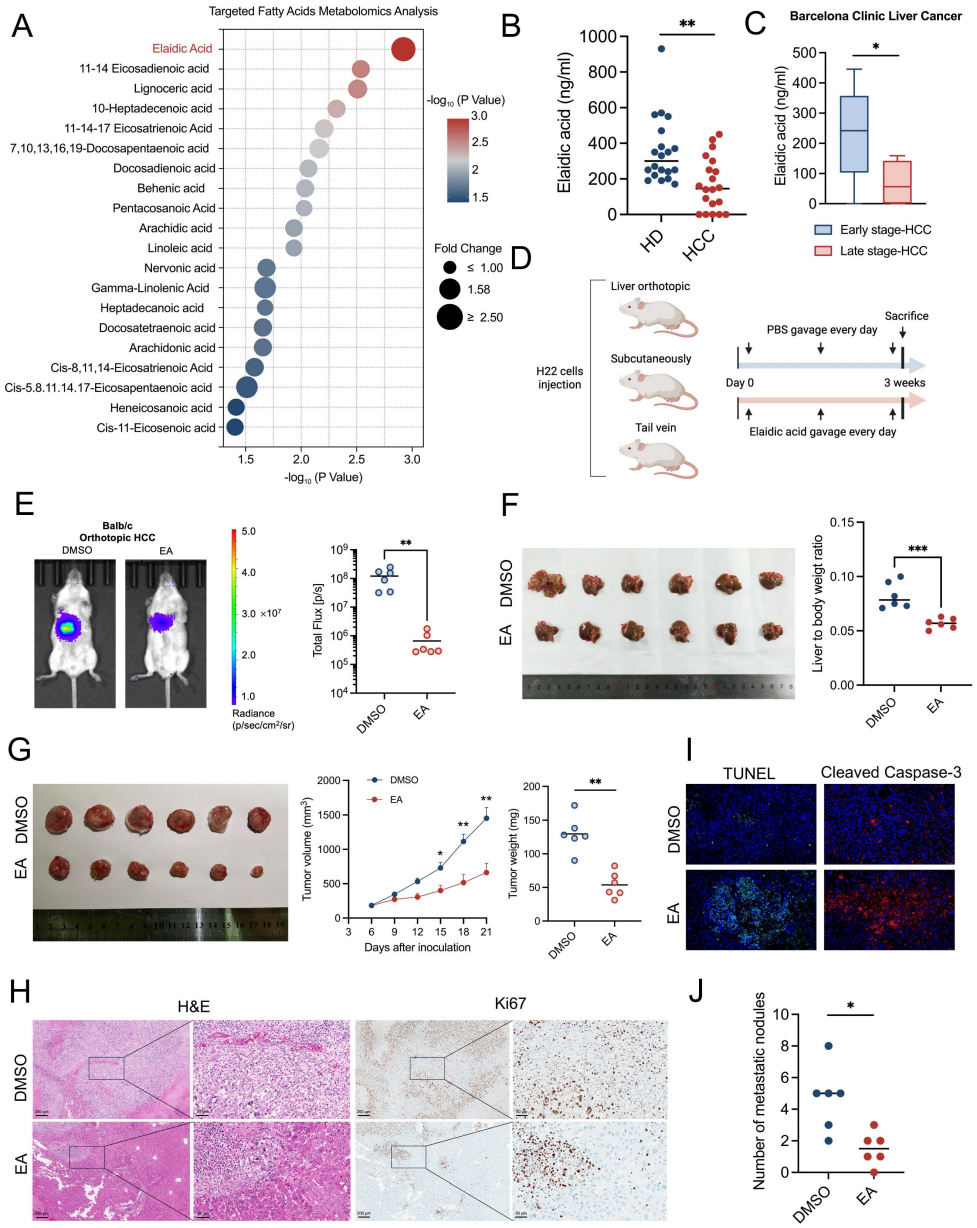
** Dietary elaidic acid suppresses HCC *in vivo*.** (A) Targeted fatty acid metabolomics showing the 20 types of fatty acids with the most significant reduction in plasma concentration of healthy individuals compared to HCC patients. (B) Concentration of EA in healthy donors and HCC patients. (C) Concentration of EA in the early stage of HCC and the late stage of HCC based on the Barcelona Clinic Liver Cancer staging. (D) Schematic graph demonstrating the experimental design of the establishment of HCC murine models and the gavage of EA (n=6 per group). (E) Tumor burden was visualized by IVIS at 3 weeks after liver orthotopic injection of H22 cells. Dot plot showing the fluorescence intensity of mice with different treatments (n=6 per group). Tumor burden was reduced in mice treated with EA. (F) Representative images and liver-to-body weight ratio of liver treated with DMSO or EA (n=6 per group). (G) Representative images and tumor volume changes (mm³) of subcutaneous H22 tumors from mice treated with EA (n=6 per group). (H) Macroscopic pictures and representative H&E and Ki67 images of liver tumors. Scale bars: 200 μm. (I) Representative immunofluorescence figure of TUNEL and cleaved caspase-3 in liver tumor tissue from the DMSO and EA gavaged groups. (J) Tumor burden was visualized by IVIS at 3 weeks after tail vein injection of H22 cells. Dot plot showing the fluorescence intensity of mice with different treatments. (n=6 per group). HD, healthy donors; HCC, hepatocellular carcinoma; DMSO, dimethyl sulfoxide; EA, elaidic acid; IVIS, *in vivo* imaging system; H&E, hematoxylin and eosin staining; TUNEL, Terminal deoxynucleotidyl transferase dUTP Nick End Labeling. Significance was calculated using an unpaired t-test. Significant P values were indicated, and error bars were shown as mean ± sd. *p<0.05, **p<0.01, ***p<0.001.

Targeted fatty acid metabolomics analysis suggested that EA showed the most significant decrease in the plasma of HCC patients compared to healthy individuals among the 44 types of fatty acids (Figures 1A, 1B). Moreover, the concentration of EA in the late stage of HCC patients is markedly higher than in the early stage of HCC patients, based on the Barcelona Clinic Liver Cancer staging (Figure 1C) and China liver cancer staging (Figure S1A). To investigate the effect of EA on liver cancer, we established three kinds of murine models, including orthotopic liver cancer, subcutaneous xenograft tumor, and tail vein injection mouse models, and administered EA to them (Figure 1D). In the orthotopic liver cancer model, we quantified the tumor growth of mice by an *in vivo* imaging system (IVIS). Results showed that the tumor burden of HCC mice significantly decreased after the administration of EA compared to the DMSO-treated group (Figure 1E). In addition, the volume of orthotopic liver tumors and the liver-to-body weight ratio of HCC mice significantly decreased after administration of EA (Figure 1F). As a kind of known trans-fatty acids, EA is associated with cardiovascular diseases. We detected the concentration of several cardiovascular toxicity indexs, including total cholesterol, triglyceride, lactate dehydrogenase, creatine kinase, high-density lipoprotein cholesterol, and low-density lipoprotein cholesterol in the serum of HCC mice from the DMSO and EA gavaged groups, respectively. Results showed that there is no significant difference between the DMSO group and the EA group (Figures S1B, S1C). In subcutaneous xenograft mice, the EA-treated group consistently showed slower tumor growth with reduced sizes and weights of the excised tumors (Figure 1G). Additionally, tumor cells following EA treatment were less Ki67-positive in IHC staining and more widely distributed in H&E staining (Figure 1H). Furthermore, the expression of TUNEL and cleaved caspase 3 of mouse liver tumors is markedly higher in the EA group compared to the DMSO group (Figure 1I). In the third mouse model established by injecting H22 cells through the tail vein, we quantified the tumor burden of the whole body and lung by IVIS and counted the percentage of mice with or without lung metastasis in two groups. Results showed that EA attenuated the metastasis of lung cancer in HCC mice (Figures 1J, S1D-S1G). These findings all corroborate the idea that dietary EA inhibited HCC *in vivo*.

Considering EA is the trans isomer of OA (oleic acid, C18:1 cis), we intend to clarify whether the observed anti-tumor effects are specific to the trans isomer (EA) or also attributable to its cis isomer. Then we administered DMSO, OA, or EA to the orthotopic HCC mice by gavage. Results revealed that OA did not inhibit the growth of liver cancer compared with the DMSO group. This suggests that the biological effects of EA may be configuration-dependent. (Figures S1H, S1I).

### Result 2: Gut microbiota contributes to elaidic acid-induced HCC suppression

**Figure 2:**
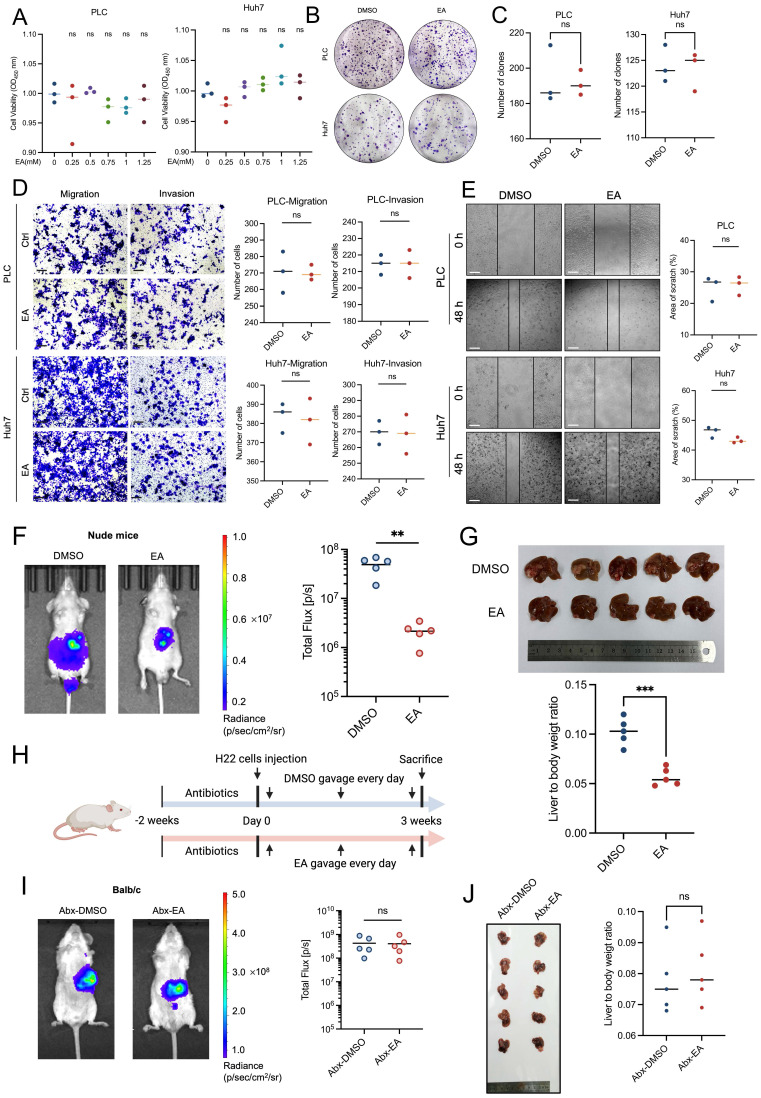
** Gut microbiota contributes to elaidic acid-induced HCC suppression.** (A) Cell proliferation of PLC and Huh7 cells after 24-h treatment with a series of concentrations of EA. (B-C) Colony formation of HCC cells under EA treatment. (D) The migration and invasion ability of EA-treated HCC cells. Scale bars: 20 μm. (E) The wound healing ability of HCC cells treated with EA. The effect of EA was evaluated by the percentage of healing area. Scale bars: 50 μm. (F) Tumor burden of nude mice treated with DMSO or EA was visualized by IVIS. Dot plot showing the fluorescence intensity of mice with different treatments (n=5 per group). (G) Representative images and liver-to-body weight ratio of livers with different treatments (n=5 per group). (H) Schematic graph demonstrating the experimental design of the mouse model treated with antibiotics. (n=6 per group). (I) The tumor burden of antibiotic-treated HCC mice was visualized by IVIS at 3 weeks after gavage of EA. Dot plot showing the fluorescence intensity of mice with different treatments (n=5 per group). (J) Representative images of liver tumors (n=5 per group). Liver-to-body weight ratio of the mice treated with DMSO and EA. PLC, human liver cancer cell line; Huh7, human liver cancer cell line; DMSO, dimethyl sulfoxide; EA, elaidic acid; Abx, antibiotics. Significance was calculated using an unpaired t-test. Significant P values were indicated, and error bars were shown as mean ± sd. **p<0.01; ***p<0.001; ns, not significant.

First of all, we treated PLC and Huh7 with varying concentrations of EA (including 0, 0.25, 0.50, 0.75, 1.00, and 1.25 mM) for 24 h in order to confirm the anti-HCC action of EA. However, the cell viability of HCC cells has no significant change under the treatment of diverse concentrations of EA (Figure 2A).

In addition, we expanded the experiment to include higher concentration gradients, including 2.50 and 5.00 mM EA for 24 h. Results showed that the two concentrations of EA reach the cytotoxicity threshold (Figures S2A, S2B). Moreover, we added additional time points, including 48 and 72 h. Results revealed that the growth of PLC and Huh7 was suppressed markedly when treated with EA for 72 h, which fully characterizes dose-time-effect interactions (Figures S2C, S2D). In this article, rather than the cytotoxic effects of EA, we mainly intend to investigate how EA suppresses HCC *in vivo*.

In addition, the colony formation experiment results revealed that EA could not suppress the proliferation ability of PLC and Huh7 (Figures 2B, 2C). Consistently, the transwell and wound healing experiments showed that EA could not attenuate the migration and invasion ability of PLC and Huh7 (Figures 2D, 2E), which is contrary to the *in vivo* experiments and inspires us to explore the cause of the phenomenon.

While EA did not affect tumor cells *in vitro*, it inhibited tumor growth *in vivo*, suggesting that factors existing solely *in vivo* exerted an inhibitory effect on tumors, such as immune cell-mediated effects. We established a liver orthotopic tumor model in nude mice with immune dysfunction and administered DMSO or EA by gavage, respectively. Results showed that EA suppresses the growth of HCC in mice (Figures 2F, 2G).

Considering that intestinal flora plays a significant role within the body, we speculated about the relation between the tumor-suppressing effect of EA and gut microbiota and conducted antibiotic treatment on mice. The gut microbes in the mouse intestines were depleted by treatment of an antibiotic cocktail (Abx) including 1 g/L metronidazole, 1 g/L ampicillin, 1 g/L neomycin, and 0.5 g/L vancomycin in water for drinking during two weeks. Then we injected H22 cells into mice livers to establish Abx-HCC mouse models (Figure 2H). After oral gavage of DMSO or EA for 3 weeks, IVIS revealed that there was no marked difference between the two groups (Figure 2I). Then the mice were dissected, and we observed that the size of orthotopic liver tumors did not significantly differ between the groups treated with EA and DMSO, and the liver-to-body weight ratio showed the same trend (Figure 2J). At the same time, EA-treated tumor cells were comparable to the DMSO group in H&E staining as well as Ki67 IHC staining (Figures S2E, S2F). To investigate whether antibiotics non-specifically accelerate tumor progression, which could artifactually cause EA to appear ineffective, we established recovery groups. We conducted gut microbiota reconstruction after antibiotic intervention, antibiotic cessation, and transplantation of fecal microbes from healthy mice. Results revealed that EA exerts significant tumor-suppression effects (Figures S2G-S2J). Therefore, the observed loss of EA's effect on HCC progression is specifically attributed to microbiota depletion rather than antibiotic-induced side effects.

### Result 3: Dietary elaidic acid alters gut microbiota and increases the abundance of *Ligilactobacillus murinus*

**Figure 3:**
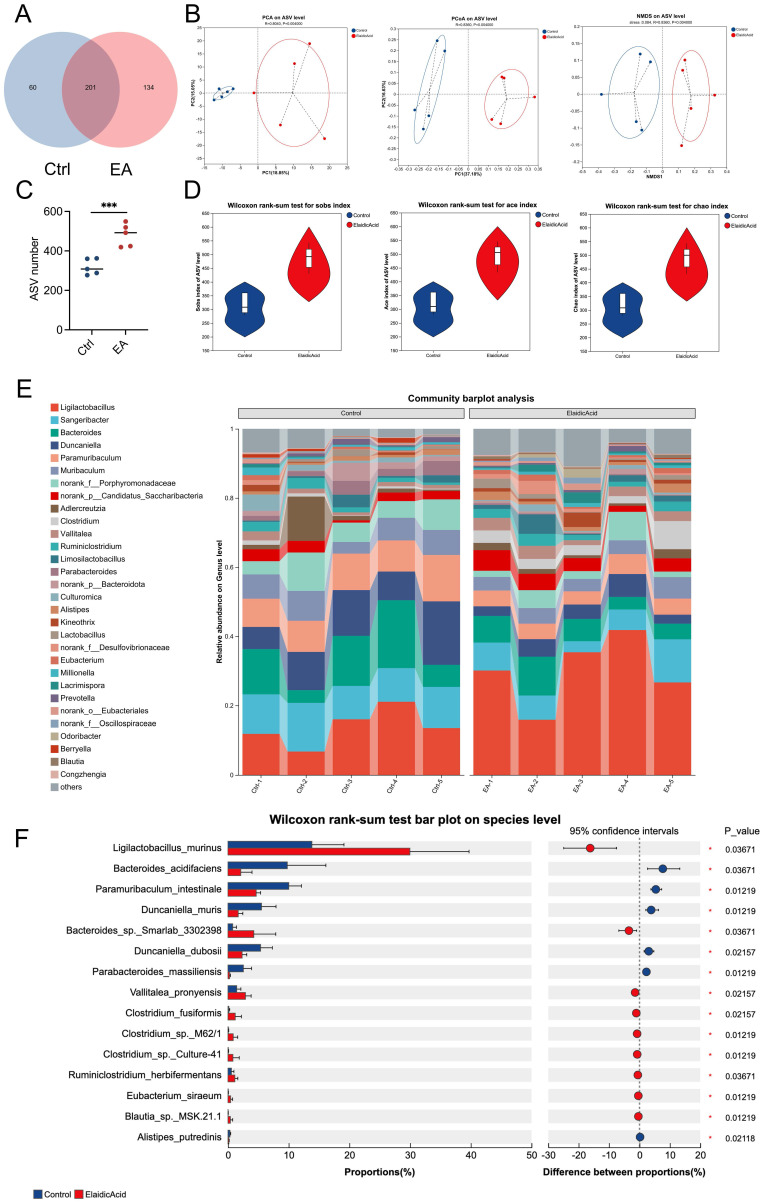
** Dietary elaidic acid alters gut microbiota and increases the abundance of *Ligilactobacillus murinus*.** (A) The blue circle represents the DMSO-treated group; the red circle represents the EA-treated group; the overlapping part indicates the species shared by the two groups, while the non-overlapping part represents the species unique to each group. The numbers indicate the corresponding number of species. (B) Principal Components Analysis, Principal Coordinate Analysis, and Non-metric Multi-dimensional Scaling Analysis on the ASV level of the intestinal flora of groups treated with DMSO or EA. (C) ASV numbers of the intestinal flora of the DMSO- or EA-treated group. (D) Wilcoxon rank-sum test for alpha diversity estimators such as Sobs, Ace, and Chao index. (E) Community barplot analysis for relative abundance of the intestinal flora between the DMSO and EA groups on the genus level. (F) Wilcoxon rank-sum test bar plot of the intestinal flora between the DMSO and EA groups on the species level. Ctrl, control group treated with DMSO; EA, elaidic acid; PCA, Principal Components Analysis; PCoA, Principal Coordinates Analysis; NMDS, Non-metric Multidimensional Scaling; ASV, Amplicon Sequence Variant; Sobs, observed index; Ace, abundance-based coverage estimator index. Significance was calculated using an unpaired t-test. Significant P values were indicated, and error bars were shown as mean ± sd. ***p<0.001.

To investigate the connection between intestinal microbiota and dietary EA consumption, we conducted 16S ribosomal RNA sequencing (16S rRNA-seq) on the feces of orthotopic liver cancer mouse models that had been administered with EA or DMSO. We found that 201 kinds of bacterial species were shared by both groups of mice (Figure 3A). After administration of EA, the Principal Coordinate Analysis (PCoA), Principal Components Analysis (PCA), and Non-metric Multi-dimensional Scaling (NMDS) analyses of the Amplicon Sequence Variant (ASV) levels of the intestinal microbiota in the mice showed significant differences between the two groups (Figure 3B). EA administration increased the ASV levels and alpha diversity, such as the species observed (Sobs) index, abundance-based coverage estimator (Ace) index, and Chao index of the intestinal microbiota in the mice (Figures 3C, 3D). In detail, the community barplot and further analysis revealed that the most significantly increased species in the intestinal microbiota is *Ligilactobacillus murinus* (*L. murinus*) (Figures 3E, 3F, S3A-S3E). These results indicate that dietary intake of EA altered gut flora and significantly increased the abundance of *L. murinus* in the intestinal tract.

### Result 4: *Ligilactobacillus murinus* suppresses the HCC growth *in vitro* and *in vivo*

Since the anti-tumor effect of EA is dependent on the intestinal microbiota, and the abundance of *L. murinus* increased most dramatically in the EA-treated group, we hypothesized that *L. murinus* plays an important role in inhibiting tumor growth.

To clarify whether *L. murinus* can directly translocate into the liver to exert a tumor-suppressing effect, we detected the abundance of *L. murinus* in the liver tissue of Abx-HCC orthotopic mice gavaged with PBS or *L. murinus*. Results showed that there is no significant difference between the two groups, and the cycle threshold of both groups in quantitative PCR assays was all above thirty-five (Figure S4A). Therefore, we speculate the *L. murinus* does not exert its tumor-suppressing effects by directly transferring to the liver.

**Figure 4:**
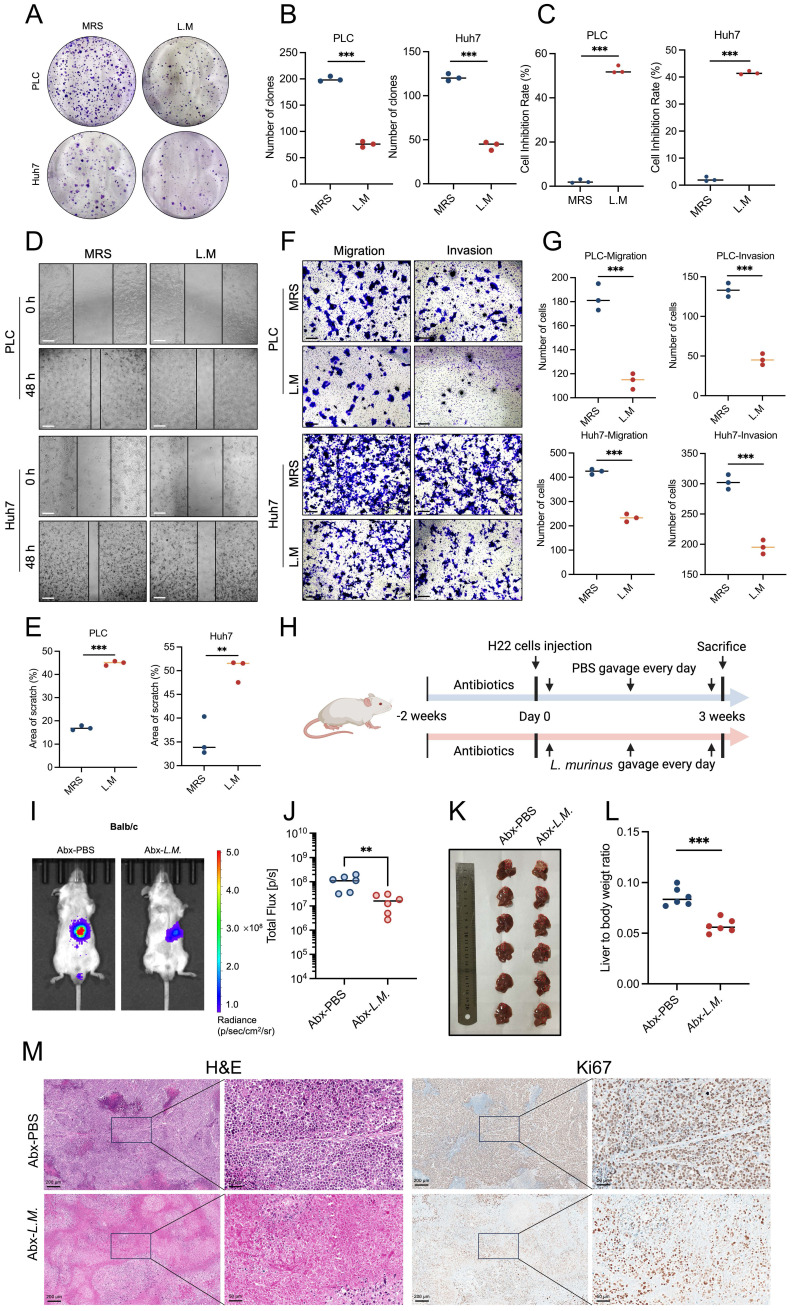
***Ligilactobacillus murinus* suppresses the HCC *in vitro* and *in vivo*.** (A-B) Colony formation of PLC and Huh7 cells after 24-h treatment with the supernatant of *L. murinus*. (C) Cell proliferation of PLC and Huh7 cells after 24-h treatment with the supernatant of *L. murinus*. (D) The wound healing ability of PLC and Huh7 cells after 24-h treatment with the supernatant of *L. murinus*. (E) The effect of the supernatant of *L. murinus* was evaluated by the percentage of healing area. Scale bars: 50 μm. (F-G) The migration and invasion ability of PLC and Huh7 cells after 24-h treatment with the supernatant of *L. murinus*. Scale bars: 20 μm. (H) Schematic graph demonstrating the experimental design of HCC mice treated with *L. murinus*. (n=6 per group). (I) Tumor burden was visualized by IVIS at 3 weeks after antibiotic treatment, liver orthotopic injection of H22 cells, and treatment of *L. murinus*. (J) Dot plot showing the average radiance intensity of mice with different treatments (n=6 per group). Tumor burden was decreased after treatment with *L. murinus*. (K) Representative images of liver tumors. (L) Liver-to-body weight ratio of the mice treated with *L. murinus*. (M) Macroscopic pictures and representative H&E and Ki-67 images of liver tumors. Scale bars: 200 μm. MRS, deMan, Rogosa, and Sharpe medium; *L.M.*, *Ligilactobacillus murinus*; PLC, human liver cancer cell line; Huh7, human liver cancer cell line; Abx, antibiotics. Significance was calculated using an unpaired t-test. Significant P values were indicated, and error bars were shown as mean ± sd. **p<0.01, ***p<0.001.

Considering that bacterial-derived metabolites play a critical role in tumor progression, we isolated the supernatant of the *L. murinus* bacteria and conducted a series of *in vitro* cell functional experiments. The results of the cell counting kit-8 (CCK-8) assay and colony formation assay showed that the supernatant of *L. murinus* significantly inhibited the proliferation of human liver cancer cell lines PLC and Huh7 (Figures 4A-4C). Similarly, liver cancer cells' capacity to migrate and invade was greatly inhibited via the wound healing and transwell experiments (Figures 4D-4G). These results suggest that *L. murinus*-derived supernatant suppresses the HCC *in vitro*.

Next, the anti-tumor activity of *L. murinu* was then evaluated by creating an orthotropic mouse model with H22 cells (Figure 4H). When antibiotic-treated (Abx) HCC mice were subjected to a daily gavage of *L. murinus*, results showed that the tumor burden of mice with intestinal flora transplantation was significantly reduced (Figure 4I-M). In conclusion, intestinal *L. murinus* inhibits the growth of HCC both *in vivo* and *in vitro*.

### Result 5: *Ligilactobacillus murinus*-derived spermidine suppresses HCC *in vitro*

**Figure 5:**
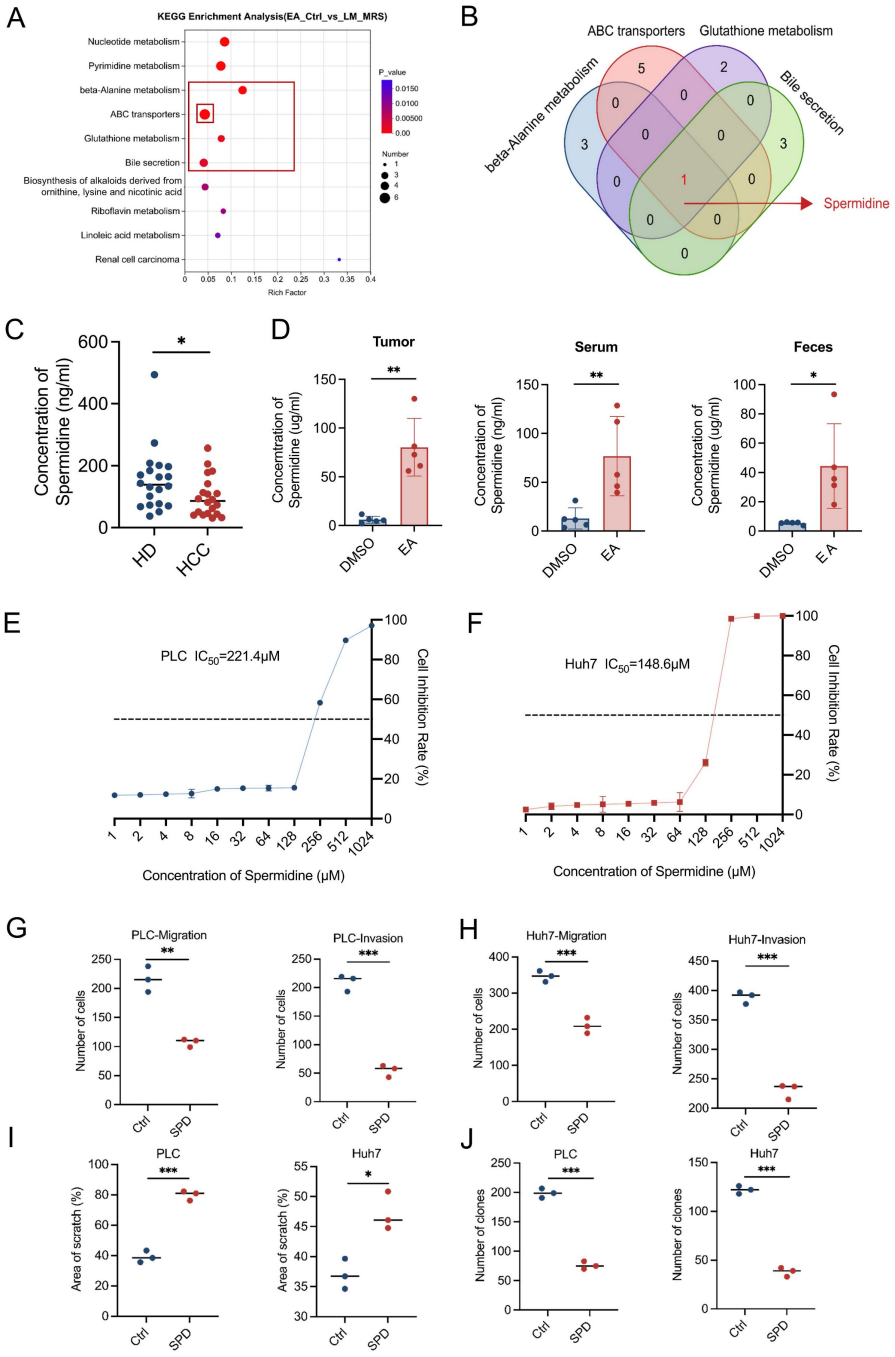
***Ligilactobacillus murinus*-derived spermidine suppresses HCC *in vitro*.** (A) KEGG metabolic pathway. Differentially expressed metabolic pathways were ranked by P value, and the top 10 metabolic pathways were shown. (B) Among the top six metabolic pathways that consist of differentially expressed metabolites, four of them (β-Alanine metabolism, ABC transporters, Glutathione metabolism, and Bile secretion) all contained the same metabolite, spermidine. (C) Concentration of SPD in healthy donors and HCC patients. (D) Concentration of SPD in tumor, serum, and feces of mice with different treatments (n=5 per group). (E-F) Cell proliferation of PLC and Huh7 cells after 24-h treatment with SPD. (G-H) The migration and invasion ability of PLC and Huh7 cells after 24-h treatment with SPD. (I) The effect of SPD was evaluated by the percentage of healing area. (J) Colony formation of PLC and Huh7 cells after 24-h treatment with SPD. EA, elaidic acid; Ctrl (A), control group treated with dimethyl sulfoxide; *L.M.*, *Ligilactobacillus murinus*; MRS, deMan, Rogosa, and Sharpe medium; HD, healthy donors; HCC, hepatocellular carcinoma; DMSO, dimethyl sulfoxide; IC_50_, half maximal inhibitory concentration; Ctrl (Figures G-J), control group treated with PBS; SPD, spermidine. Significance was calculated using an unpaired t-test. Significant P values were indicated, and error bars were shown as mean ± sd. *p<0.05, **p<0.01, ***p<0.001.

To determine the specific metabolites in the supernatant of cultivation of* L. murinus* exerting a tumor-suppressing effect, we conducted untargeted metabolomics sequencing to analyze the unique differential metabolites of the feces of EA-treated HCC mice relative to the feces of DMSO-treated HCC mice, as well as the unique differential metabolites of *L. murinus* supernatant relative to de Man, Rogosa, and Sharpe (MRS) medium. By taking the intersection of the two metabolite sets, we obtain a new set that contains *L. murinus*-derived metabolites that are highly enriched in the EA-treated group. Through Kyoto Encyclopedia of Genes and Genomes (KEGG) enrichment analysis, we found that among the top six metabolic pathways consisting of differentially expressed metabolites, four of them (β-Alanine metabolism, ABC transporters, Glutathione metabolism, and Bile secretion) all contained the same metabolite, spermidine (SPD) (Figures 5A, 5B, S4B-S4G).

Then we detected the content of SPD in human serum using ELISA assays. Results showed that the concentration of SPD in HCC patients is markedly lower than in healthy humans (Figure 5C). We also performed quantitative ELISA assessments of SPD levels in feces, serum, and tumor tissues of PBS- or *L. murinus*-gavaged orthotopic HCC mice. Results revealed that the levels of SPD in feces, serum, and tumor tissues of EA-treated HCC mice are markedly higher than in DMSO-treated mice (Figure 5D).

To verify whether SPD suppresses HCC, we added SPD to the culture supernatant of tumor cells and conducted a series of cell functional experiments *in vitro*. The CCK-8 assay showed that SPD significantly inhibited the proliferation of human liver cancer cell lines PLC and Huh7 (Figures 5E, 5F). Moreover, the wound healing and transwell assays revealed that SPD significantly inhibited the migration and invasion abilities of PLC and Huh7 (Figures 5G-5I, S4I, S4J). Consistently, the colony formation experiments indicated that SPD significantly suppressed the *in vitro* proliferation ability of PLC and Huh7 (Figures 5J, S4H). In conclusion, *L. murinus*-derived SPD suppresses HCC *in vitro*.

### Result 6: *Ligilactobacillus murinus*-derived spermidine suppresses HCC *in vivo*

**Figure 6:**
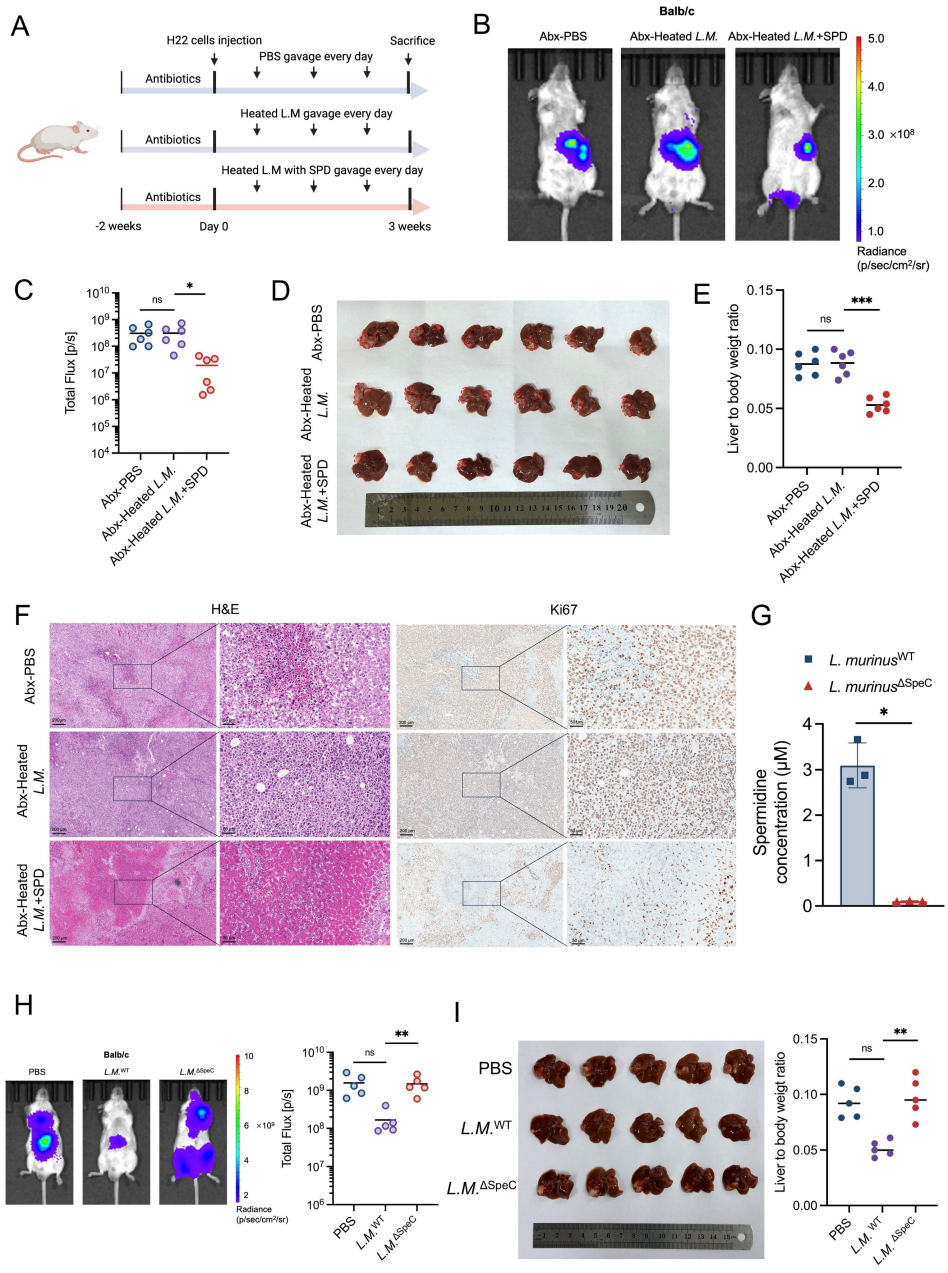
***Ligilactobacillus murinus*-derived spermidine suppresses HCC *in vivo*.** (A) Schematic graph demonstrating the experimental design (n=6 per group). (B) Tumor burden was visualized by IVIS at 3 weeks of antibiotic-treated HCC mice after gavage of heated *L.M.* with or without SPD. (C) Dot plot showing the average radiance intensity of mice with different treatments (n=6 per group). (D) Representative images of liver tumors from antibiotic-treated HCC mice after gavage of heated *L.M.* with or without SPD. (E) Liver-to-body weight ratio of the mice, antibiotics-treated HCC mice after gavage of heated *L.M.* with or without SPD. (F) Macroscopic pictures and representative H&E and Ki-67 images of liver tumors. Scale bars: 200 μm. (G) Concentration of SPD released from wild-type *L. murinus* and ornithine decarboxylase-depleted *L. murinus*. (H) Tumor burden of orthotopic HCC mice with different treatments was visualized by IVIS. Dot plot showing the fluorescence intensity of the mouse liver tumors (n=5 per group). (I) Representative images and liver-to-body weight ratio of liver with different treatments (n=5 per group). Abx, antibiotics; *L.M., Ligilactobacillus murinus*; SPD, spermidine; HCC, hepatocellular carcinoma; IVIS, *in vivo* imaging system; H&E, hematoxylin and eosin staining. Significance was calculated using an unpaired t-test. Significant P values were indicated, and error bars were shown as mean ± sd. *p<0.05, **p<0.01, ***p<0.001; ns, not significant.

To suppress the production of SPD derived from *L. murinus*, we heated *L. murinus* in the 100°C metal bath for 30 min to establish heat-killed strains and administered the bacteria intragastrically to HCC mice with or without SPD (Figure 6A). Results of IVIS demonstrated that the tumor burden of HCC mice in the heated-inactivated *L. murinus* bacteria with SPD combined-treated group was significantly reduced compared to the PBS-treated group and the heat-inactivated *L. murinus* bacteria-treated group (Figures 6B, 6C), which was consistent with the volume and weight of the orthotopic tumor tissues (Figures 6D, 6E). In addition, H&E and Ki67 immunohistochemical staining showed that the pathological changes of liver cancer in SPD-treated mice were attenuated. On the contrary, the pathological changes of liver cancer in mice with intestinal transplantation of heat-inactivated *L. murinus* were comparable to the control group (Figure 6F).

To explore whether EA treatment promotes the enrichment of other SPD-producing bacterial species, we constructed the ornithine decarboxylase-deleted *L. murinus* strain by targeting SpeC genes using the suicide vector PNZ5319 18, which has been reported to cause a severe reduction in SPD production. And we measured the concentration of SPD in the supernatant of *L. murinus* ^WT^ or *L. murinus* ^∆SpeC^ cultured *in vitro* using ELISA assays. Results showed that the production of SPD of *L. murinus* ^∆SpeC^ has been markedly suppressed (Figure 6G). Then, the orthotopic HCC mouse models were orally administered PBS, *L. murinus* ^∆SpeC^, and *L. murinus* ^WT^, respectively. As we didn't use antibiotics, there are other gut microbiota in the intestines of the three groups of HCC mice. Results showed that compared to the PBS group, *L. murinus* ^WT^ significantly suppresses the growth of HCC. However, tumor burden in the* L. murinus* ^∆SpeC^ group and the PBS group was nearly the same (Figure 6H). Results revealed that when the ability to produce SPD of *L. murinus* was severely inhibited, the growth of HCC in mice was almost fully recovered (Figure 6I). To some extent, *L. murinus* is the most important gut microbiota that inhibits the progression of HCC. And we measured the blood cell count and important indicators related to liver function from the plasma and serum of mice and found that the concentration of SPD ingested by the HCC mice was insufficient to cause toxic damage to them (Figure S5A-S5M). These results indicate that SPD, as a metabolite of *L. murinus*, exerts a tumor-suppressing effect.

### Result 7: SPD induces the apoptosis of HCC by activating the p38 MAPK signaling pathway

**Figure 7:**
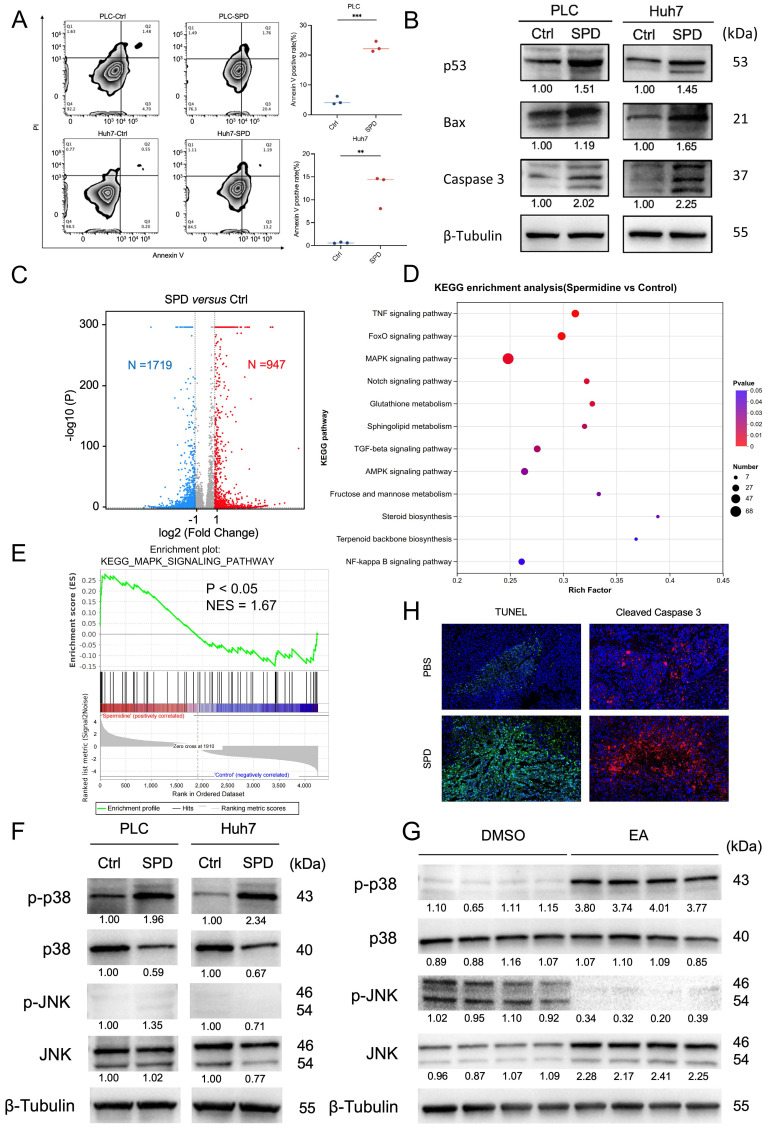
** SPD induces the apoptosis of HCC by activating the p38 MAPK signaling pathway.** (A) The effect of SPD on HCC cell apoptosis. Representative images from the flow cytometry were shown. The percentage of annexin V-positive HCC cells was estimated. (B) Western blot of apoptosis and proliferation biomarkers related to cell proliferation and apoptosis. (C) RNA sequencing analysis of human liver cancer Huh7 cells treated with SPD (n = 3) or PBS (n = 3), the volcano plot revealed differentially expressed genes. (D) KEGG pathway enrichment analysis of RNA-seq. (E) GSEA analysis of RNA-seq. (F) Western blot of p-p38, p38, p-JNK, and JNK in human liver cancer cells PLC and Huh7. (G) Western blot of p-p38, p38, p-JNK, and JNK in tumors from HCC mice treated with DMSO and EA. (H) Representative immunofluorescence figure of TUNEL and cleaved caspase-3 in liver tumor tissue from the PBS and SPD gavaged groups. PLC, human liver cancer cell line; Huh7, human liver cancer cell line; Ctrl, control group treated with DMSO; SPD, spermidine; KEGG, Kyoto Encyclopedia of Genes and Genomes; RNA-seq, RNA sequencing; GSEA, Gene set enrichment analysis; p-p38, phosphorylated p38 MAPK; p38, mitogen-activated protein kinase 14; p-JNK, phospho-c-Jun N-terminal kinases; JNK, c-Jun N-terminal kinases; DMSO, dimethyl sulfoxide; EA, elaidic acid. Significance was calculated using an unpaired t-test. Significant P values were indicated, and error bars were shown as mean ± sd. **p<0.01, ***p<0.001.

To clarify whether SPD inhibits HCC growth by inducing tumor cell death, we conducted flow cytometry analysis after the PLC and Huh7 cells were treated with SPD for 24 h. The experiment revealed that the apoptosis rates of PLC and Huh7 were significantly increased after treatment with SPD (Figure 7A). Western blot experiments showed that the expressions of tumor protein 53 (p53), Bcl-2-associated X protein (Bax), and cysteine-requiring aspartate protease 3 (Caspase 3) in PLC and Huh7 treated with SPD significantly increased (Figure 7B).

To illuminate the downstream signaling mechanism involved in SPD-induced HCC suppression, we conducted RNA-seq of the SPD-treated HCC cell line Huh7 and of those from the control group. Volcano plots revealed that 947 genes were up-regulated, and 1719 genes were down-regulated in SPD-treated HCC (Figure 7C). KEGG pathway enrichment analysis revealed that several pathways, including TNF, Foxo, and the Mitogen-Activated Protein Kinase (MAPK) pathway, were upregulated in the SPD-treated group, with the MAPK signaling pathway exhibiting the most differentially expressed genes (Figure 7D). Enrichment score revealed that MAPK signaling was significantly activated by SPD (Figure 7E). Considering p38 and JNK in the MAPK family are associated with apoptosis, we detected the expression and phosphorylation of the protein in human liver cancer cells and liver tumors from orthotopic HCC mice by western blot. Results showed that SPD significantly promoted the phosphorylation of p38 MAPK in both human and mouse samples in the SPD group, consistently (Figures 7F, 7G). Through the staining of TUNEL and cleaved caspase-3 in mouse liver tumor tissues, we found the apoptosis rate of HCC of the SPD-group mice was higher than the PBS-group (Figure 7H). In conclusion, SPD activated the phosphorylation of p38 MAPK in HCC.

**Figure 8:**
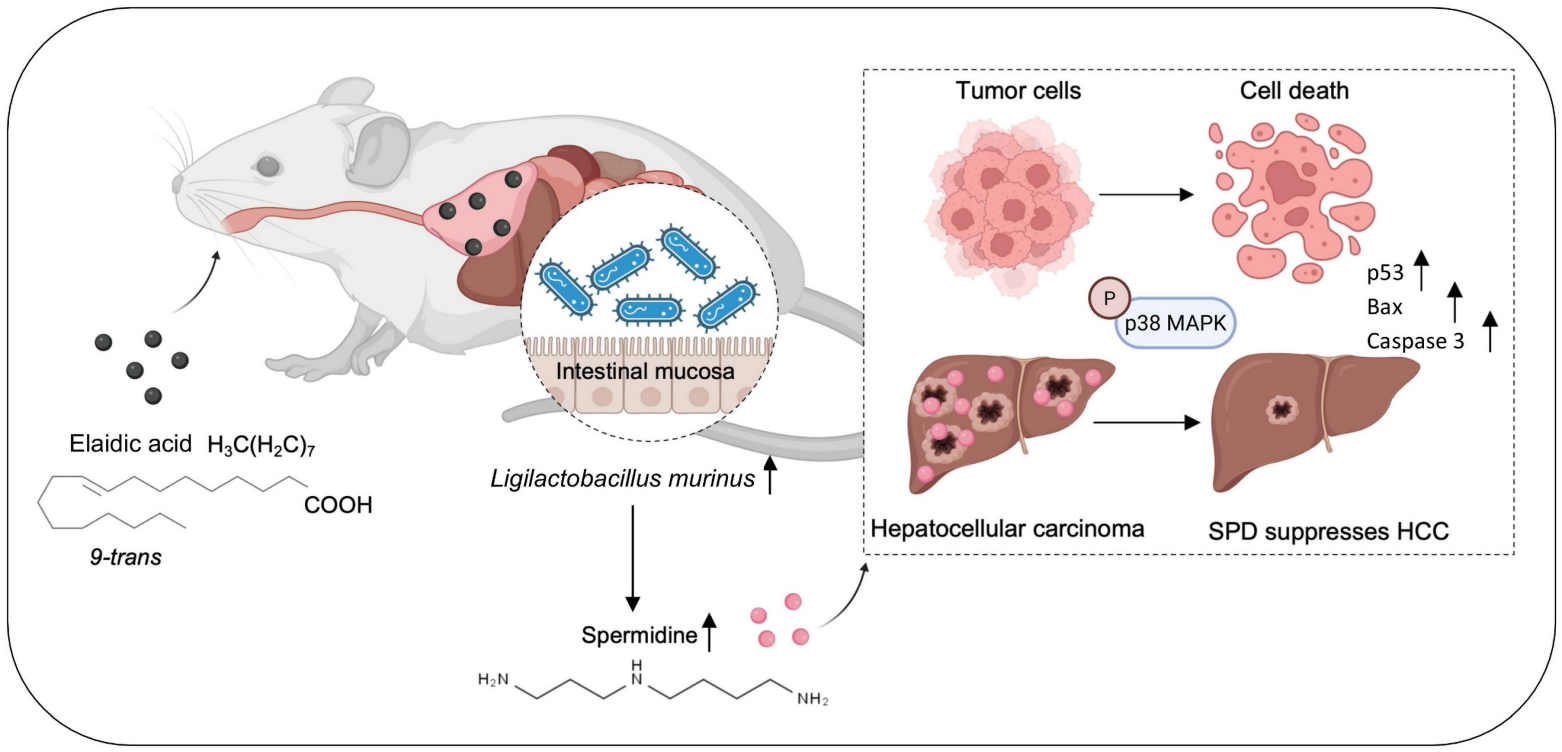
** EA suppresses HCC growth through modulating the production of intestinal *L. murinus*-derived SPD.** SPD activates the phosphorylation of p38 MAPK in HCC and induces apoptosis.

In summary, we identified *L. murinus* as the bacterium suppressing HCC tumorigenesis. The intake of EA increases the abundance of *L. murinus* and the production of *L. murinus*-derived SPD in mouse models. We elucidated that the *L. murinus*-derived metabolite SPD activated the downstream activation of the p38 MAPK signaling pathway and induced apoptosis in HCC. Our study thus provides evidence of *L. murinus* as a promising probiotic for HCC treatment (Figure 8).

## 4. Discussion

In this study, we found that EA showed the most significant decrease in the plasma of HCC patients compared to healthy individuals among 44 types of fatty acids through targeted fatty acid metabolomics analysis. Therefore, we intend to illuminate whether EA exerts anti-tumor effects on HCC. Firstly, we examined the anti-tumor effects of EA by xenograft and orthotopic HCC mouse models. However, *in vitro* assays suggested that EA-treated HCCs are comparable with DMSO-treated HCCs, which inspires us to explore the reason for the phenomenon. Considering that gut microbiota has a significant part in the organism, we use a cocktail of antibiotics to deplete the intestinal microbiota of mice. After establishing orthotopic HCC mouse models and supplementing the diet with EA, we found that EA loses anti-tumor function in the Abx group, which provides an initial verification of the relation between EA and gut microbiota.

To explore the mechanism, we studied the effect of EA treatment on gut microbiota by 16S rRNA-seq. Results demonstrated that *L. murinus* abundance was enhanced most significantly in the group treated with EA as opposed to the group treated with DMSO. Then we separated the supernatant of the *L. murinus* and added it to Huh7 and PLC cells to assess the change in tumor cells. A series of *in vitro* assays revealed that *L. murinus* suppresses HCC. Then we established the mouse model with intestinal microbiota transplantation, which further confirmed the anti-tumor effect of *L. murinus*.

To further explore which substance in the supernatant of the *L. murinus* suppresses HCC, we conducted metabolomic sequencing analysis of the feces from EA-treated and DMSO-treated HCC, along with MRS medium and the supernatant of the *L. murinus*. Results revealed that among the top six metabolic pathways consisting of differentially expressed metabolites, four of them all contained the same metabolite, SPD, which is derived from *L. murinus*. Cell function assays were conducted to delineate tumor cell suppression. The HCC mice were then subjected to SPD treatment, and the tumor burden was quantified using IVIS. These assays revealed that SPD suppresses HCC both *in vitro* and *in vivo*. Moreover, we further found that SPD enhances the apoptosis of liver tumor cells by contributing to the expression of p53, Bax, and Caspase 3 through flow cytometry and western blot. However, gavaging heat-inactivated *L. murinus*, which fails to secrete SPD, reversed tumor suppression effects. Taken together, *L. murinus* enrichment caused by EA gavage is protective against HCC through microbial metabolite secretion.

Metabolites derived from gut microbiota are important links between the development of cancer and the gut microbiota 19. As important natural products, they alter the tumor microenvironment and influence the functionality of immune cells and cancer cells 20. Certain microbial metabolites exhibit direct carcinogenic properties. Specifically, colibactin, produced by commensal *pks*^+^ *Escherichia coli* in the gut, functions as a DNA alkylating agent and induces genomic instability within human intestinal epithelial cells, a process that can culminate in the development of colorectal cancer 21. However, certain microbiota-derived metabolites exhibit oncostatic properties. The gut bacterium *Lactobacillus reuteri* produces reuterin, which inhibits ribosome biogenesis and induces protein oxidation to target the progression of colorectal cancer 22. Additionally, several metabolites of microorganisms have both anticancer and carcinogenic properties. The functional impact of metabolites is context-dependent, critically influenced by the diversity of resident cancer cells and the local metabolite concentration 23. At physiological concentrations, SCFAs demonstrate a potent capacity to suppress colon cancer progression 24. On the contrary, SCFAs are particularly elevated. Elevated SCFA dosages have the potential to surpass the host tolerance threshold and accelerate the development of HCC linked to non-alcoholic fatty liver disease 25. Furthermore, the proliferation, apoptosis, and metastasis of tumor cells are influenced by the regulation of several signaling pathways by microbial metabolites, primarily involving the MAPK, PI3K/Akt, NFκB, and Wnt signaling pathways 10. Furthermore, the effects of cancer treatment can be significantly improved by using microbial metabolites in radiotherapy 26, 27, chemotherapy 28, 29, and immunotherapy 30, 31.

Previous studies revealed that SPD increases lifespan and inhibits HCC and hepatic fibrosis via triggering MAP1S-mediated autophagy 32. Moreover, SPD was shown to stimulate mitochondrial trifunctional protein and enhance anti-cancer immunity in murine models 33. In our study, we showed that SPD induces the apoptosis of live cancer cells. Moreover, it remains unclear whether SPD can modulate the function of immune cells. In addition, we have not yet elucidated the intricate mechanism of how SPD exerts the anti-tumor effect, which is worth further investigation. Furthermore, we have not fully clarified whether SPD is suitable for the treatment of patients with HCC or other malignancies.

In addition, there are still some areas for improvement that are worth discussing. As shown in the cardiovascular toxicity index values of DMSO- or EA-treated HCC mice, although there was no statistically significant difference in the six main indicators of the mice in the two groups, mice in the EA intervention group showed a slight increase in creatine kinase levels overall. In addition, one mouse in the EA group had a relatively high lactate dehydrogenase value. Therefore, the intervention dose of EA could be considered to be lowered to better avoid the cardiovascular risks that EA may bring.

Furthermore, in the *in vitro* part of EA experiments, we have space for optimization in terms of concentration and time parameters. As for the reason why the highest concentration of 1.25 mM was chosen for the *in vitro* experiment, according to a study published in *Food and Chemical Toxicology* 34*,* the IC_50_ of EA against HepG2 cells was around 1.0 mM in cell viability experiments. However, in our experiments, 1.0 mM EA failed to inhibit the growth of PLC and Huh7. Therefore, we set a higher concentration of 1.25 mM above 1.0 mM to test whether EA can inhibit cell growth *in vitro*, and the experimental results proved that it still couldn't. In the supplementary materials, we expanded the range of the experiment to include higher concentrations, including 2.50 mM and 5.00 mM. At the same intervention time, we found that concentrations of 2.50 mM and 5.00 mM had a significant inhibitory effect on cell growth. Therefore, we speculate that EA may also act directly on cells at higher concentrations. In addition, we added more time points of action, including 48 and 72 hours. The results showed that EA may also act directly on cells when the *in vitro* action time reaches 72 hours, indicating that time dynamics are very important for cell viability experiments. The above are our limitations in our experimental design, and the concentration range and dose-time effect of the intervention should be fully considered when conducting *in vitro* experiments so as to provide a more solid foundation for research.

Moreover, it is indeed necessary to compare the bidirectional effects across cancers. A previous study demonstrated that treating with EA has a significant metastatic capability of colorectal cancer cells 8. On the contrary, a recent study revealed that EA is possible to impede tumor advancement and improve the efficacy of PD-1 treatment through dietary supplementation 9. In our study, we found that EA suppresses HCC growth through modulating the production of intestinal *L. murinus*-derived SPD via activating the p38 MAPK signaling pathway. Therefore, EA plays different types of roles in different types of tumors.

In addition, the human patient sample size of our research is relatively small, which is our shortcoming. Additional models, such as patient-derived models, are the direction of our future efforts. As for the clinical translation value of EA in the treatment of liver cancer, we tested the concentration of EA in the blood of early and late patients and found that EA was associated with the stage of HCC patients, so EA can predict tumor progression of HCC. In addition, we tested LDL, HDL, and other indicators in the serum of mice treated with EA and found that the dose we used was not toxic, suggesting that it may be used in clinical practice in the future. However, before anything enters the clinic, various necessary clinical trials, such as humanized animal models, must be carried out, which is our next research direction to promote the further transformation of experimental conclusions.

Furthermore, it is a great question whether direct administration of SPD or supplementation with *L. murinus* as a probiotic might present more practical effects. As for direct supplementation with SPD, firstly, natural foods have SPD, but it is not easy to eat enough SPD. Secondly, whether long-term intake of high doses of SPD will have side effects on the body is worth further exploring through experiments. As for direct supplementation with *L. murinus*, immunocompromised patients may aggravate the condition by causing opportunistic infections or abnormal proliferation of bacterial flora after taking bacterial solutions. Secondly, patients with intestinal perforation, obstruction, and severe inflammatory bowel diseases who take bacterial fluid directly may cause the transplanted flora to enter the bloodstream through the broken mucosa and cause sepsis. In addition, EA accounts for 80-90% of total trans fatty acids in foods. We can change the intake of EA by adjusting the diet, which has the potential to be a safer and more feasible treatment strategy. These three treatment strategies need to be explored in further clinical trials, which is an important part of our future work.

## Supplementary Material

Supplementary materials and methods, figures.

Supplementary table 1: the concentration of fatty acids in plasma of healthy individuals and HCC patients analyzed by targeted fatty acids metabolomics.

Supplementary table 2: the tumor volume of xenograft subcutaneous mice treated with DMSO or EA.

## Abbreviations

HCC: hepatocellular carcinoma
TFA: Trans fatty acid
EA: elaidic acid
OA: oleic acid
*L.M.*: *Ligilactobacillus murinus*
SPD: spermidine
SPF: specific pathogen-free
PBS: phosphate-buffered saline
IVIS: *in vivo* imaging system
PLC: human liver cancer cell line
Huh7: human liver cancer cell line
OD: optical density
FBS: fetal bovine serum
CCK-8: Cell Counting Kit-8
IC-50: half maximum inhibitory concentration
H&E: Hematoxylin and eosin
HD: healthy donors
DMSO: dimethyl sulfoxide
TUNEL: Terminal deoxynucleotidyl transferase dUTP Nick End Labeling
Abx: antibiotics
MRS: deMan, Rogosa, and Sharpe medium
Sobs: species observed index
Lefse: linear discriminant analysis effect size
LDA: Linear discriminant analysis
PCA: Principal Components Analysis
PCoA: Principal Coordinates Analysis
PLS-DA: Partial Least Squares Discriminant Analysis
NMDS: Non-metric Multidimensional Scaling
KEGG: The enrichment of Kyoto Encyclopedia of Genes and Genomes
RNA-seq: RNA sequencing
GSEA: Gene set enrichment analysis
p-p38: phosphorylated p38 MAPK
p38: mitogen-activated protein kinase 14
p-JNK: phospho-c-Jun N-terminal kinases
JNK: c-Jun N-terminal kinases
