# Supplementary material for: Elaidic acid suppresses hepatocellular carcinoma growth through modulating the production of intestinal Ligilactobacillus murinus-derived spermidine
Source: Int J Biol Sci. 2026 Jan 14;22(3):1542–59. doi: 10.7150/ijbs.122392 (PMC12839069; doi:10.7150/ijbs.122392)

Supplementary materials and methods

Fecal microbial transplantation (FMT)

Firstly, the gut microbiota in murine intestines were depleted through the administration of an antibiotic cocktail comprising 1 g/L metronidazole, 1 g/L ampicillin, 1 g/L neomycin, and 0.5 g/L vancomycin in water for drinking over two weeks. Then collected mouse feces, cultured them after resuspension with sterile saline, and counted the colonies to ensure the successful establishment of mice with gut flora removal. Next, collected fresh feces from healthy mice (without antibiotic treatment within 8 weeks before feces collection). After being dissolved in 10 g/L sterile saline, the feces were centrifuged at 2000 rpm for 5 min. The supernatants of stools were administered to the antibiotic-treated mice (200 μl/mouse) for 2 weeks to recover gut microbiota [4]. Specifically, the feces from mice were collected and cultured after resuspension with sterile saline. Counted the colonies to ensure the successful reconstruction of gut flora in mice compared to healthy mice.

Construction of knockout plasmid pNZ5319R

The construction process of the knockout plasmid can be briefly stated as follows [18]: Left and right homologous arms (speC/FL and speC/FR, corresponding to the upstream and downstream sequences of the speC/F gene) were amplified by PCR using speC/FLF, speC/FLR, speC/FRF, and speC/FRR primers with the Lactobacillus

genome as a template. The speC/FL and speC/FR fragments were separately ligated upstream of the lox66 and downstream of the lox71 sites on pNZ5319 to obtain the knockout plasmid pNZ5319R. The procedure was as follows: speC/FL was inserted between the PmeI and XhoI sites of pNZ5319 through restriction digestion and ligation to generate plasmid pNZ5319L. The speC/FR fragment was inserted between the SmaI and BglII sites of pNZ5319L, resulting in the final knockout plasmid pNZ5319R. The above process is completed by Shanghai Genechem Company (Shanghai, China).

Preparation of *L. murinus* $^{\Delta SpeC}$

The enzyme cut sites at the ends of the upstream and downstream regions of suicide plasmid pNZ5319 were selected, and approximately 1000 kb fragments were cut and ligated, respectively, to the target gene. The recombinant plasmid pNZ5319R containing the upstream and downstream segments of the target gene was obtained and then transformed into competent *L. murinus* by electrotransfection. After two homologous recombinations, the target gene was replaced in the Lactobacillus genome to obtain the SpeC/F mutant [18].

RNA-seq

We treated the human liver cancer cell line Huh7 with SPD or PBS for 24 h. Collected the cells and washed with PBS twice. Cells were suspended in TRIzol

solvent. The library construction and sequencing were done by Majorbio Bio-Pharm Technology Co., Ltd. (Shanghai, China).

ELISA

The concentration of SPD was detected using the Spermidine ELISA kit (Wuhan Fine Biotech Co., Ltd., Wuhan, China).

Supplementary Figures

Supplemental Figure 1. EA within the safe dose range is associated with the inhibition of HCC, related to Figure 1.

Supplemental Figure 2. Antibiotics do not interfere with the tumor-suppressing effect of EA, related to Figure 2.

Supplemental Figure 3. 16S rRNA sequencing of feces from orthotopic HCC mice treated with DMSO or EA, related to Figure 3.

Supplemental Figure 4. *Ligilactobacillus murinus*-derived spermidine suppresses HCC *in vitro*, related to Figure 5.

Supplemental Figure 5. The toxicological results, including the blood indicators and representative H&E images of organs from mice, related to Figure 6.

Supplementary Tables

Supplementary Table 1. The plasma fatty acid concentration of healthy individuals and HCC patients was analyzed by targeted fatty acid metabolomics.

Supplementary Table 2. The tumor volume of xenograft subcutaneous mice treated with DMSO or EA.

Supplemental Figure Legends

Supplemental Figure 1. EA within the safe dose range is associated with the inhibition of HCC, related to Figure 1.

(A) Concentration of EA in the early stage of HCC and the late stage of HCC based on China Liver Cancer Staging.

(B-C) Concentration of total cholesterol, triglyceride, lactate dehydrogenase, creatine kinase, high-density lipoprotein cholesterol, and low-density lipoprotein cholesterol in serum of HCC mice from the DMSO and EA gavaged groups, respectively.

(D) Tumor burden was visualized by IVIS at 3 weeks after tail vein injection of H22 cells. (n=6 per group).

(E) Dot plot showing the number of metastasis nodules from mice treated with DMSO or EA.

(F) Tumor burden of lungs was visualized by IVIS at 3 weeks after injection of H22 cells through the tail vein (n=6 per group). Dot plot showing the fluorescence intensity of lungs from mice treated with DMSO or EA.

(G) Metastasis rate of mice treated with DMSO or EA.

(H) Tumor burden of HCC orthotopic mice treated with DMSO, OA, or EA was visualized by IVIS. Dot plot showing the fluorescence intensity of mice with different treatments (n=5 per group).

(I) Representative images and liver-to-body weight ratio of liver with different treatments (n=5 per group).

CNLC, China Liver Cancer Staging; DMSO, dimethyl sulfoxide; EA, elaidic acid; OA, oleic acid. Significance was calculated using an unpaired t-test. Significant P values were indicated, and error bars were shown as mean ± sd. *p<0.05, **p<0.01; ns, not significant.

Supplemental Figure 2. Antibiotics do not interfere with the tumor-suppressing effect of EA, related to Figure 2.

(A-B) Cell viability of PLC and Huh7 cells treated with EA at 0, 1.25, 2.50, or 5.00 mM for 24 h.

(C-D) Cell viability of PLC and Huh7 cells treated with EA at 1.25 mM for different time points.

(E-F) Macroscopic pictures and representative H&E and Ki-67 images of liver tumors.

(G) Antibiotic-treated HCC orthotopic mice gavaged with DMSO or EA after fecal microbiota transplantation from healthy mice.

(H) Tumor burden was visualized by IVIS.

(I) Dot plot showing the fluorescence intensity of mice with different treatments (n=5 per group).

(J) Representative images and liver-to-body weight ratio of liver with different treatments (n=5 per group).

Abx, antibiotics; DMSO, dimethyl sulfoxide; EA, elaidic acid; H&E, hematoxylin and eosin staining. Significance was calculated using an unpaired t-test. Significant P values were indicated, and error bars were shown as mean ± sd. *p<0.05, **p<0.01; ns, not significant.

Supplemental Figure 3. 16S rRNA sequencing of feces from orthotopic HCC mice treated with DMSO or EA, related to Figure 3.

(A) Heatmap showing sample distances on the species level.

(B) Lefse Bar showing LDA score of gut microbiota.

(C) Community barplot analysis for relative abundance of the intestinal flora between the DMSO and EA groups on the family level.

(D) Community barplot analysis for relative abundance of the intestinal flora between the DMSO and EA groups on the species level.

(E) Hierarchical clustering tree on the species level of gut microbiota.

Ctrl, control group treated with dimethyl sulfoxide; EA, elaidic acid; HCC, hepatocellular carcinoma; DMSO, dimethyl sulfoxide; EA, elaidic acid; Lefse, linear discriminant analysis effect size; LDA, linear discriminant analysis.

Supplemental Figure 4. *Ligilactobacillus murinus*-derived spermidine suppresses HCC *in vitro*, related to Figure 5.

(A) Abundance of *L. murinus* in liver tissue of Abx-HCC orthotopic mice gavaged with PBS or *L. murinus*. The plot showed the cycle threshold of q-PCR of bacteria.

(B) Correlation between MRS medium and the supernatant of *L. murinus*.

(C) PCA scores of feces from orthotopic HCC mice treated with DMSO or EA.

(D) PCA scores of MRS medium and the supernatant of *L. murinus*.

(E) PLS-DA scores of feces from orthotopic HCC mice treated with DMSO or EA.

(F) PLS-DA scores of MRS medium and the supernatant of *L. murinus*.

(G) The red circle represents the differential metabolite set in the feces from the DMSO- and EA-treated groups, which contains 429 kinds of metabolites; the blue circle represents the differential metabolite set in the MRS medium group and the supernatant of the *L. murinus group,* which contains 285 kinds of metabolites; the intersection of the two sets was established as a new set, representing the differential metabolites secreted by *L. murinus* in the intestinal tract of EA-treated mice, which contains 247 kinds of metabolites.

(H) Colony formation of PLC and Huh7 cells after 24-h treatment with PBS or SPD.

(I) The migration and invasion ability of PLC and Huh7 cells after 24-h treatment with PBS or SPD. Scale bars: 20 μm.

(J) The wound healing ability of PLC and Huh7 cells after 24-h treatment with PBS or SPD. Scale bars: 50 μm.

Abx, antibiotics; *L.M., Ligilactobacillus murinus*; Control (Figures C-E) group treated with dimethyl sulfoxide; HCC, hepatocellular carcinoma; DMSO, dimethyl sulfoxide;

EA, elaidic acid; MRS, deMan, Rogosa, and Sharpe medium; PCA, Principal Components Analysis; PLS-DA, Partial Least Squares Discriminant Analysis; Ctrl (Figures I, J) group treated with PBS; SPD, spermidine; ns, not significant.

Supplemental Figure 5. The toxicological results, including the blood indicators and representative H&E images of organs from mice, related to Figure 6.

(A-D) Indicators from the plasma of PBS- or SPD-treated mice.

(E-L) Indicators from the serum of PBS- or SPD-treated mice.

(M) Representative H&E images of the liver, spleen, lung, brain, kidney, and heart of PBS- or SPD-treated mice. Scale bars: 200 μm.

H&E, hematoxylin and eosin staining; SPD, spermidine; WBC, white blood cell; RBC, red blood cell; HGB, Hemoglobin; PLT, platelet; DBIL, direct bilirubin; ALT, alanine aminotransferase; AST, aspartate aminotransferase; CHE, cholinesterase; ALBP, adipocyte lipid-binding protein; TP, total protein; UA, urine acid; UN, Urea Nitrogen. Significance was calculated using an unpaired t-test. Significant P values were indicated, and error bars were shown as mean ± sd. ns, not significant.

Supplemental Figure 1

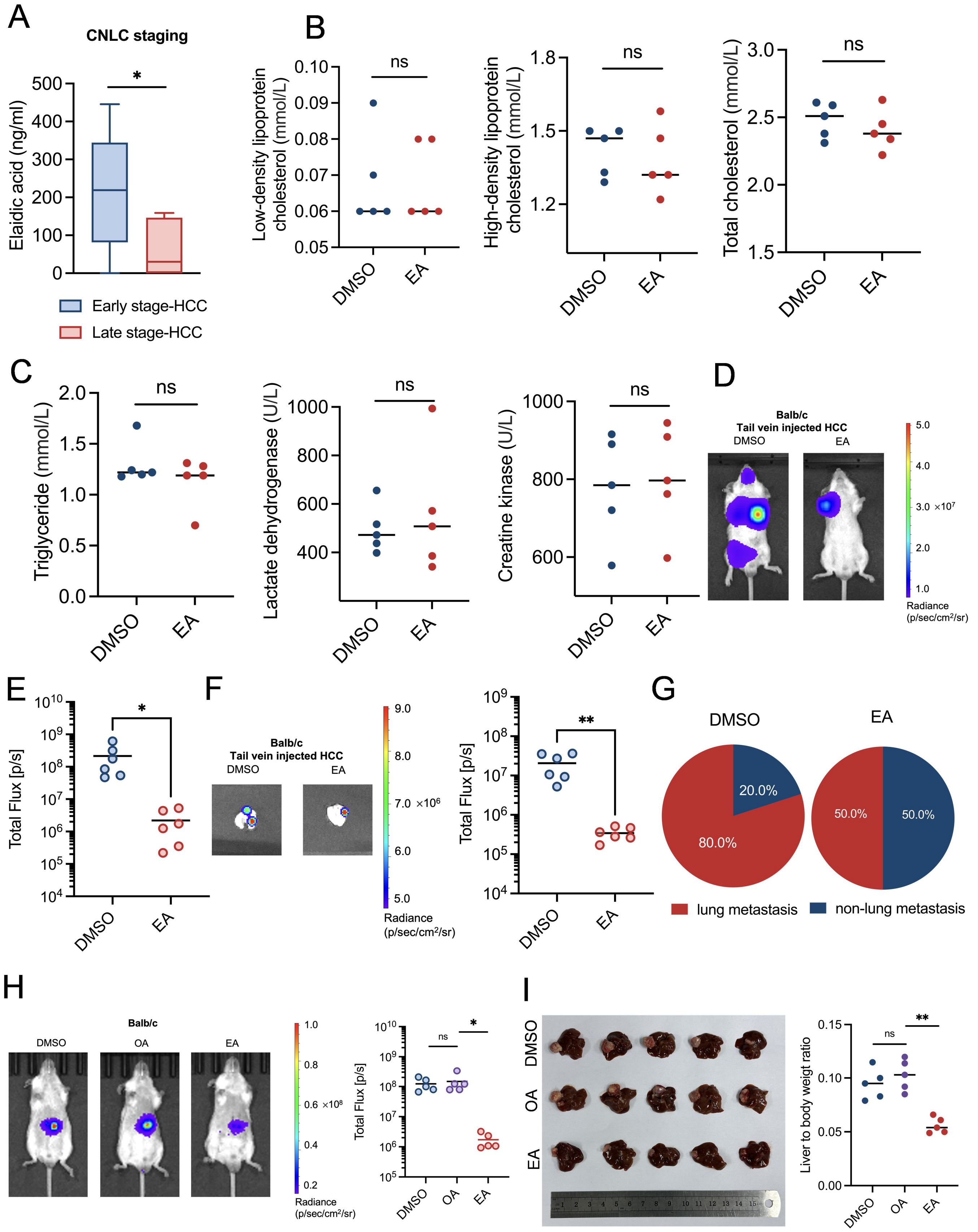

Supplemental Figure 2

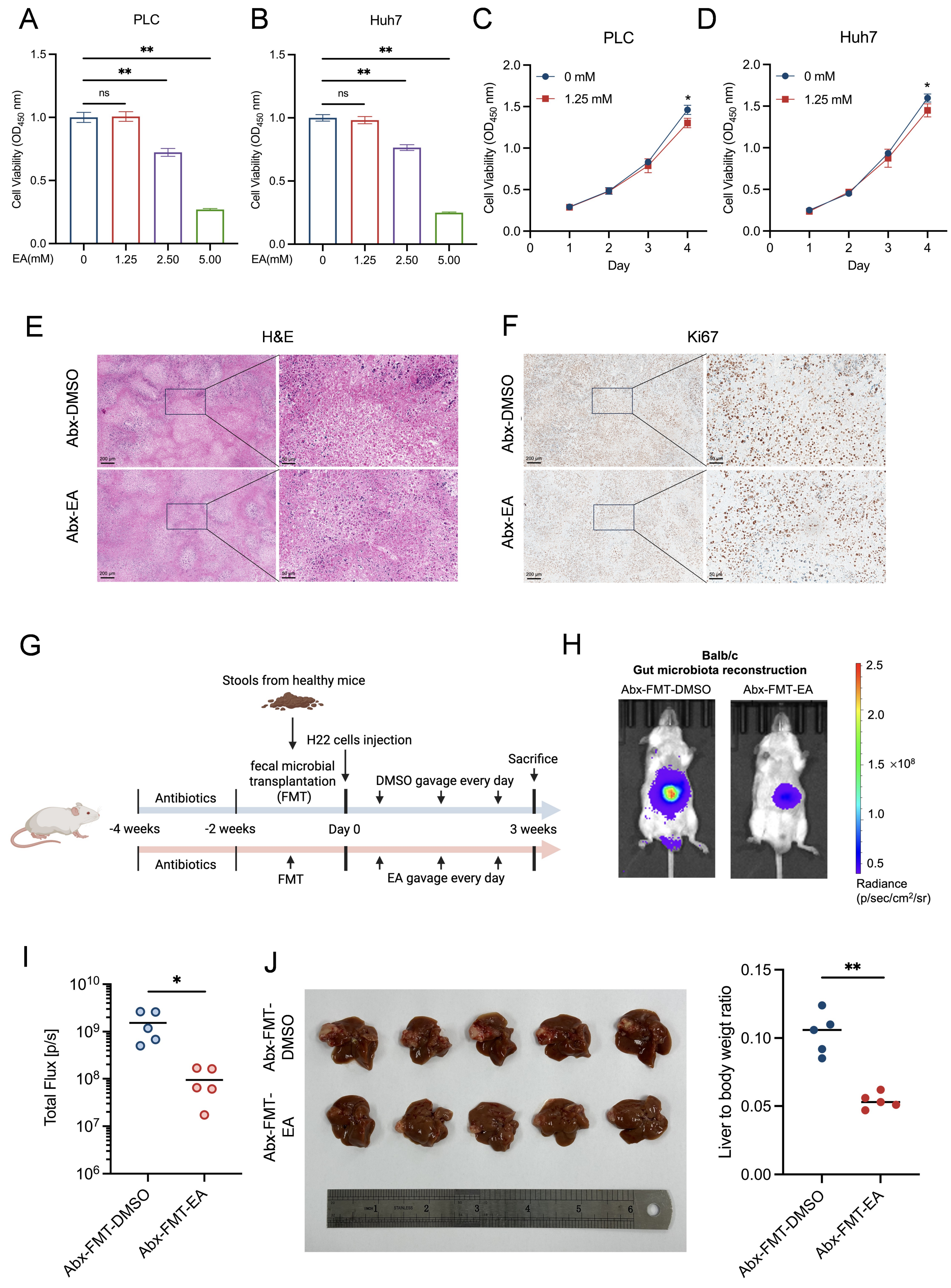

Supplemental Figure 3

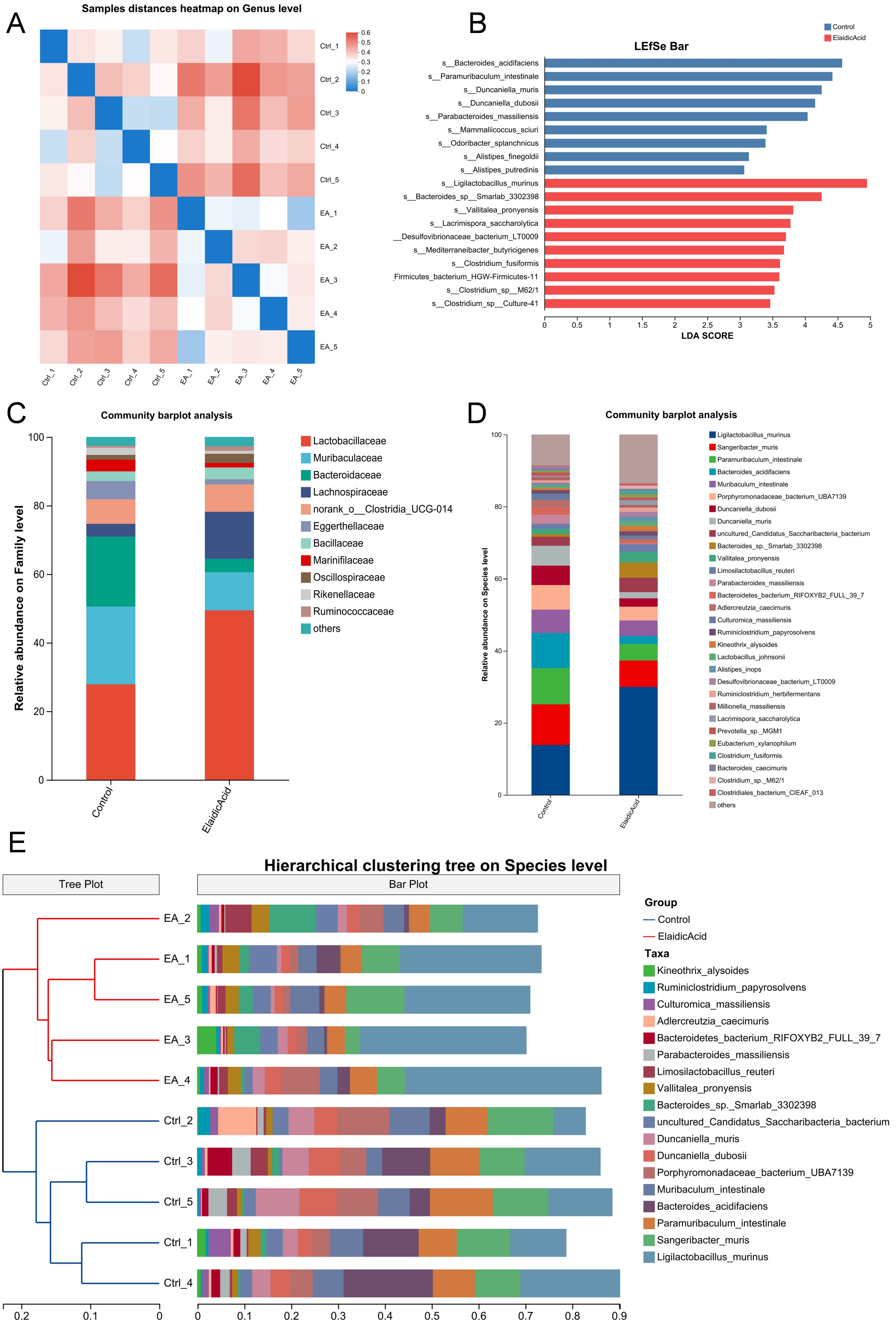

Supplemental Figure 4

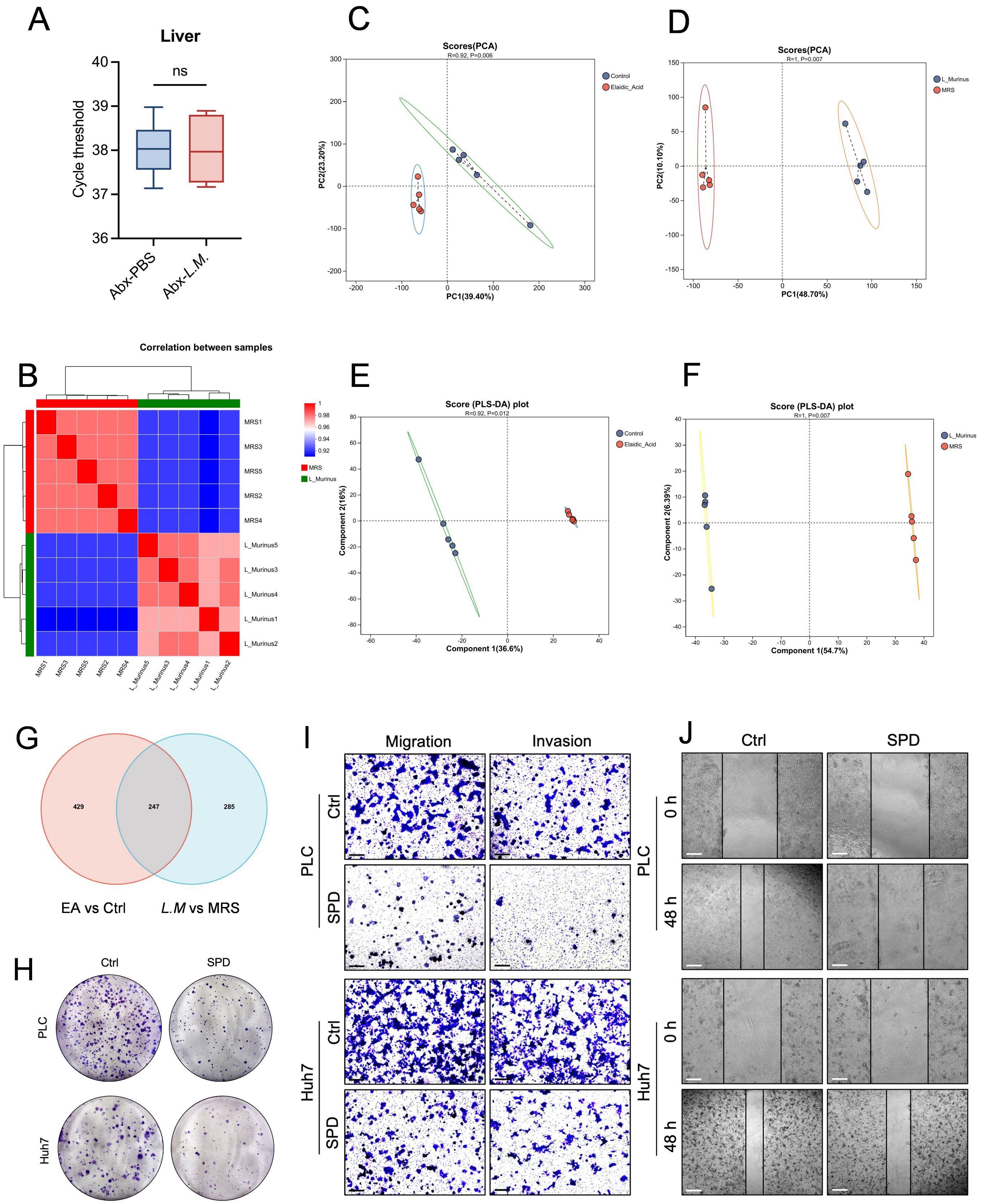

Supplemental Figure 5

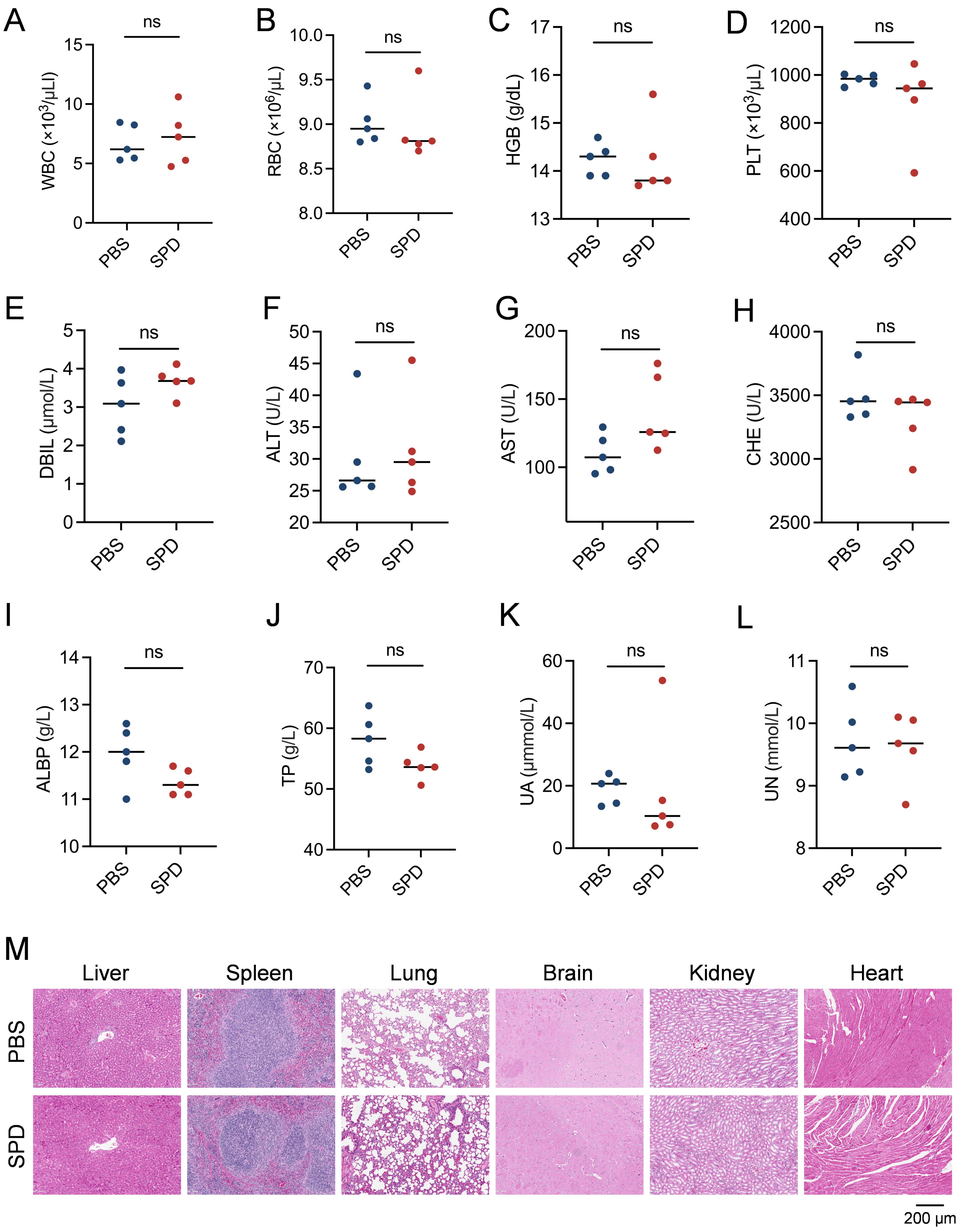

Supplement: Supplementary file 1 — Supplementary materials and methods, figures. [file ijbsv22p1542s1.pdf]
